# Detrital Carbonate Minerals in Earth's Element Cycles

**DOI:** 10.1029/2021GB007231

**Published:** 2022-05-17

**Authors:** Gerrit Müller, Janine Börker, Appy Sluijs, Jack J. Middelburg

**Affiliations:** ^1^ Department of Earth Sciences Utrecht University Utrecht The Netherlands; ^2^ Institute for Geology CEN (Center for Earth System Research and Sustainability) Universität Hamburg Hamburg Germany

**Keywords:** detrital carbonate, particulate inorganic carbon, alkalinity, calcium, biogeochemical cycling, river sediment

## Abstract

We investigate if the commonly neglected riverine detrital carbonate fluxes might reconciliate several chemical mass balances of the global ocean. Particulate inorganic carbon (PIC) concentrations in riverine suspended sediments, that is, carbon contained by these detrital carbonate minerals, were quantified at the basin and global scale. Our approach is based on globally representative data sets of riverine suspended sediment composition, catchment properties, and a two‐step regression procedure. The present‐day global riverine PIC flux is estimated at 3.1 ± 0.3 Tmol C/y (13% of total inorganic carbon export and 4% of total carbon export) with a flux‐weighted mean concentration of 0.26 ± 0.03 wt%. The flux prior to damming was 4.1 ± 0.5 Tmol C/y. PIC fluxes are concentrated in limestone‐rich, rather dry and mountainous catchments of large rivers near Arabia, South East Asia, and Europe with 2.2 Tmol C/y (67.6%) discharged between 15°N and 45°N. Greenlandic and Antarctic meltwater discharge and ice‐rafting additionally contribute 0.8 ± 0.3 Tmol C/y. This amount of detrital carbonate minerals annually discharged into the ocean implies a significant contribution of calcium (∼4.75 Tmol Ca/y) and alkalinity fluxes (∼10 Tmol (eq)/y) to marine mass balances and moderate inputs of strontium (∼5 Gmol Sr/y) based on undisturbed riverine and cryospheric inputs and a dolomite/calcite ratio of 0.1. Magnesium fluxes (∼0.25 Tmol Mg/y), mostly hosted by less‐soluble dolomite, are rather negligible. These unaccounted fluxes help in elucidating respective marine mass balances and potentially alter conclusions based on these budgets.

## Graphical abstract

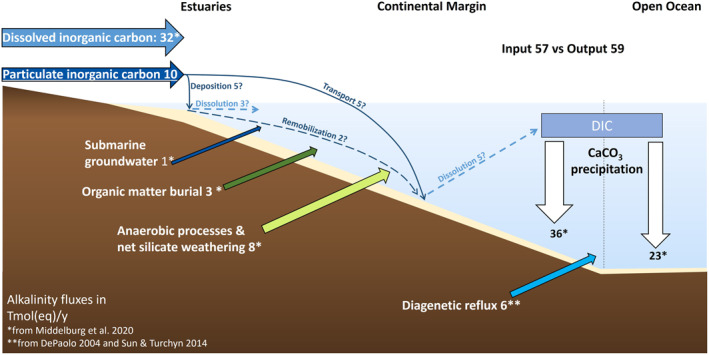

1

**Key Points**

- The present‐day riverine detrital carbonate flux from land to sea is 3.1 ± 0.3 Tmol C/y (= 0.037 Pg C/y)
- Associated calcium, alkalinity, and strontium contribute significantly to their global biogeochemical cycles
- Damming reduced the riverine Particulate inorganic carbon flux by 25% (from naturally 4.1 ± 0.5 Tmol C/y = 0.049 Pg C/y)

## 2 Introduction

Erosion and weathering of Earth's surface not only shape landscapes but also influence the global carbon cycle, thereby maintaining the habitability of our planet (Berner et al., 1983; Ebelmen, 1845; Ferrier & West, 2017; Penman et al., 2020; Urey, 1952; West et al., 2005). Oceanic mass balances of carbon (C) and biogeochemically coupled elements provide a powerful tool to investigate these processes and their role in the Earth system globally and over longer time scales (classically >100 ka) (Berner & Berner, 2012; Dickens, 2001; Krabbenhöft et al., 2010; Tipper et al., 2010). They also allow quantification of hardly measurable processes, such as global rates of marine carbonate burial or hydrothermal activity (Shalev et al., 2019; Tipper et al., 2006; van der Ploeg et al., 2019). However, some of the most prominent and most frequently considered budgets presented in that context remain unbalanced and/or highly debated, such as those of Ca, Mg, Sr, and alkalinity (Berner & Berner, 1987, 2012; Gislason et al., 2006; Jones et al., 2012; Krabbenhöft et al., 2010; Lebrato et al., 2020; Milliman, 1993; Tipper et al., 2006, 2010). This is usually explained by disequilibrium, that is, the present state strongly differs from average Pleistocene conditions, by proposing a variety of smaller‐scale marine processes and/or by invoking yet unaccounted input fluxes (Krabbenhöft et al., 2010; Middelburg et al., 2020; Milliman, 1993; Shalev et al., 2019; Tipper et al., 2010).

For most marine mass balances, riverine dissolved loads are traditionally considered as the only major input term, reflecting the catchment‐integrated result of chemical rock weathering as transported by the Earth‐spanning fluvial networks (Berner & Berner, 1987, 2012). Some authors recognized submarine groundwater discharge as another important flux to the ocean with a probable magnitude of 0.7%–6% of the global river discharge (Mayfield et al., 2021; Milliman, 1993; Zhou et al., 2019). In addition to these dissolved inputs, it is generally accepted that organic and biogenic riverine particles exert major control on the biogeochemical cycling of carbon (C), nitrogen (N) and phosphorous (P) (Berner, 1982, 1999; Boyer & Howarth, 2008; Froelich et al., 1982; Hilton & West, 2020), and of silicon (Si) (Conley, 2002; Sutton et al., 2018). Moreover, the importance of ions and complexes sorbed to the surfaces of riverine sediments was highlighted (Berner et al., 1983; Tipper et al., 2021). A similar importance was proposed for particulate inorganic forms (mineral detritus) of Si (Mackenzie & Garrels, 1965; 1966), calcium (Ca) (Gislason et al., 2006), strontium (Sr) (Hong et al., 2020; Jones et al., 2012), iron (Fe) (Luo et al., 2020; Poulton & Raiswell, 2002), and other elements (e.g., Abbott et al., 2019; Jeandel et al., 2011) based on experimental and field‐measured element release rates. Recently, based on a limited data set, Middelburg et al. (2020) suggested that riverine particulate inorganic carbon (PIC) fluxes to the ocean may be about 1/3 of riverine dissolved inorganic carbon (DIC) fluxes and that the ocean alkalinity budget is close to balance when this is considered an additional alkalinity input. While basaltic minerals, ashes, and glasses of volcanic origin are currently considered to be the major host minerals of particulate Ca and Sr fluxes (Gislason et al., 2006; Jones et al., 2012; Torres et al., 2020), significant riverine PIC fluxes would imply substantial additional Ca, Sr, and Mg delivery in particulate forms.

Carbonate dissolution and recrystallization are well known to occur in estuaries (Aller, 1982; Gattuso et al., 1998; Santos et al., 2019) and the ocean (Krumins et al., 2013; Milliman, 1974; Sulpis et al., 2017), providing evidence for the (partial) release of Ca, Mg, Sr, inorganic C (IC), and alkalinity from detrital sources to the oceanic inventories. Dissolution of PIC in the ocean could thus represent a major missing term in oceanic mass balances, potentially altering the conclusions deduced from those budgets (e.g., Berner & Berner, 2012; Krabbenhöft et al., 2010; Paytan et al., 2021; Tipper et al., 2006, 2010). Notably, recrystallization within the sediment column, that is, dissolution and direct reprecipitation, may result in an exchange of elements and isotopes between PIC and seawater (DePaolo, 2004; Fantle et al., 2010; Paytan et al., 2021).

High solubility and rapid dissolution kinetics of carbonate minerals cause the dominant mass of IC to be transported in dissolved form (Lasaga, 1984). Therefore, the significance of detrital carbonate minerals in river sediments is often neglected. However, detrital carbonates are commonly observed constituents of suspended sediments in rivers (Mackenzie & Garrels, 1966; Müller et al., 2021a) and even authigenic carbonate production in calcite‐saturated rivers is common (Grosbois et al., 2001; Kempe & Emeis, 1985; Négrel & Grosbois, 1999). Such authigenic carbonate formation on land represents a (temporary) sink of weathering‐derived cations, alkalinity, and carbon with implications for the location of gas exchange and for global mass balancing (Rovan et al., 2021; Zhao et al., 2016). Sr‐isotopic constraints suggest that 30%–50% of the carbonate minerals within the Gulf of Lyon sediments are detrital, even more during glacial periods (Pasquier et al., 2019). Additionally, the isotopic composition of carbonates from turbidites in the Bengal fan, one of the largest sediment dispersal systems on earth (Mouyen et al., 2018), suggests a mixture of biogenic (>85 wt%), detrital (up to 10 wt%), and diagenetic (1.2–4 wt%) origin (France‐Lanord et al., 2018). This indicates that PIC delivery may indeed be a relevant flux to the marine realm, but its size and dissolving fraction remain unclear because a global assessment is lacking.

We aim to better constrain these important numbers based on several approaches. First, we establish a first‐order calculation based on published average PIC and CaO concentrations and sediment fluxes. Next, we quantify PIC concentrations and fluxes globally at the basin scale, using published data sets of riverine suspended sediment and catchment characteristics, and a two‐step regression procedure involving regressive classification and symbolic regression (SR) (for details, see Section 2.2; Regression and Upscaling and Supporting Information S1). Controlling factors of the global PIC flux, human influence, and the fate of the delivered detrital carbonates in the ocean are then discussed, including implications for oceanic mass balances and carbon cycling.

## 3 Methods and Procedures

To calculate the global PIC flux, we need a gapless set of PIC concentrations and sediment fluxes of all rivers in the world, which is not realistically achievable from measurements. However, the latter can be generated using advanced models that provide suspended sediment fluxes of global rivers in space and time based on water balance and catchment properties (WBMSed 2.0, Cohen et al., 2014). The WBMSed 2.0 provides anthropogenically disturbed suspended sediment flux data as well as natural background values. These data, along with the locations of the river mouths, were taken from a compilation of the *GlobalDelta* project (Nienhuis et al., 2020). No such model is available for PIC concentrations yet. Hence, we here develop a statistical, spatially explicit model that predicts PIC concentrations from catchment properties. Modeled PIC concentrations were combined with both the natural and the anthropogenically disturbed suspended sediment fluxes (WBMSed 2.0) to arrive at the corresponding PIC fluxes.

Annual median PIC concentrations were calculated for all locations in the GloRiSe v1.1 database (Müller et al., 2021b) from direct measurements, mineralogical and petrographic observations, or empirically from major element composition (Supporting Information S1). The uncertainty of these concentrations was defined as the mean relative deviation of single measurements from the flux‐weighted mean of the available time series (Müller et al., 2021b). A large set of hydro‐environmental and physiographic variables were derived from the HydroBasins database (Linke et al., 2019) by spatially assigning each GloRiSe location to the corresponding subbasin (at Pfafstetter level 7). Annual averages for the upstream catchment of nine variables were selected based on correlation analysis and/or a causal link to PIC concentrations (Supporting Information S1). These variables cover topography, vegetation, hydrology, climate, and human impact (Table 1). As the carbonate in the catchment is the source of riverine PIC, a proper indication of this "source carbonate" (SC) was extracted from global maps of lithology (GLiM, Hartmann & Moosdorf, 2012), unconsolidated sediments (GUM, Börker et al., 2018), and soils (WISE, Batjes, 2012). For soils, the carbonate content was given directly, while for each rock and sediment class, a global representative estimate of the carbonate content was taken from literature (Supporting Information S1). Area‐weighted upstream averages were calculated individually for the carbonate content of GliM, GUM, and WISE in each basin. Next, these were summed and normalized to 100% to represent the SC, that is, carbonate available to be transported as PIC. All the predictor variables are summarized in Table 1. Catchments with SC < 10% were assumed to be PIC‐free as dissolution usually dominates over detrital carbonate transport in (undersaturated) rivers (see 1 Introduction).

**Table 1:** Predictor Variable Selection to Model PIC Concentrations

Topography and vegetation	Underground and humans	Climate and hydrology
Elevation	Potential source carbonate (rock, sediment, and soil)	Precipitation
Upstream catchment area	Soil organic carbon content	Temperature
Forestation	Human factor (log (hdi + gdp + nli + pop))	Extent of water bodies (rivers, lakes, and reservoirs)
Bare areas (rock, desert, tundra, and open shrub land)		

*Note.* Variables are taken from HydroBasins (Linke et al., 2019), except for the potential source carbonate, which was calculated from global soil, sediment, and lithological maps (Batjes, 2012; Börker et al., 2018; Hartmann & Moosdorf, 2012). All variables represent the upstream average of a specific HydroBasins subbasin at Pfafstetter level 7. hdi, human development index; gdp, gross domestic product; nli, night light index; pop, population count.

### 3.1 Regression and Upscaling

To estimate PIC concentrations in the remaining ∼65% of the global suspended sediment discharge, we employed a two‐step regression procedure consisting of (a) a qualitative indication of the presence of PIC in a catchment (yes/no) and (b) a quantitative regressive estimation of the PIC concentration. This two‐step procedure was necessary because PIC concentrations are not only log‐normally distributed but also frequently close or equal to zero, thus hampering the regression procedure, which is a well‐known problem in ecology (Fletcher et al., 2005).

For the qualitative model, we applied a Support Vector Machine (SVM), a standard technique from the MATLAB 2019 Machine Learning toolbox. This model was trained and forced by only five variables, because SVMs have been found to achieve better results with less variables (Kitsikoudis et al., 2013). We chose to use SC, precipitation, elevation, forestation, and human factor, covering the most diverse aspects of sources and preservation potential of PIC. PIC concentration was assumed not present if it was below 0.1 wt%, approximating the uncertainty of most measurements included in *GloRiSe*. SVM was chosen because it performed slightly better than alternative methods, such as logistic regression and ensemble techniques.

For the quantitative model, SR by means of multigene genetic programming was used, providing a fully data‐driven tool to find both the model structure and its parameters. SR was chosen because it performed better than simple linear regression or alternative machine learning techniques available (e.g., in the MATLAB Regression Learner Toolbox) in terms of both accuracy and precision. The implemented SR‐algorithm pseudorandomly creates linear combinations of (potentially nonlinear) terms, which are tested and evolved to best fit the observed PIC concentrations as assessed by the root mean squared error (*GPTIPS* 2.0, Searson et al., 2010). Thus, SR is able to cover nonlinear relationships between the variables, and its performance seems comparable to artificial neural networks, while it still results in comparably simple equations that can be related to the governing processes (Gandomi et al., 2015; Jin et al., 2019; Kitsikoudis et al., 2013). Variable selection and SR intrinsically determine the importance of individual variables for, and their direction of relationship to PIC concentrations, which we quantitatively assess using the linear correlation coefficient and coefficient of determination (R and R^2^, respectively, at *p* < 0.01) between the median result of 830 accepted Monte Carlo simulations and each variable. This method reduces biases due to multicollinearity and nonlinearity and is commonly applied to the evaluation of canonical correlations analyses (Kuylen & Verhallen, 1981).

The global riverine PIC flux is the sum of the products of sediment fluxes and PIC concentrations in each basin draining directly to the coastal ocean. For a proper reestimation and uncertainty analysis, the regression and prediction procedure was repeated 2,000 times with a (pseudo)random perturbation of sediment fluxes and PIC concentrations within the range of their respective uncertainties, including the full model derivation via SVM and SR. The final result is the mean of 830 accepted simulations that produced less than 0.3% outliers with respect to the 10% and 90% percentiles (10 of 3,364 coastal basins) and its uncertainty is the standard deviation of these models (Koehler et al., 2009). For comparison, we also provide literature‐based first‐order estimates of riverine PIC fluxes (Supporting Information S1). Because much less detailed data are available for atmospheric and cryospheric PIC contributions, these fluxes were estimated using published PIC concentrations and sediment fluxes (Supporting Information S1).

## 4 Results

**Figure 1:**
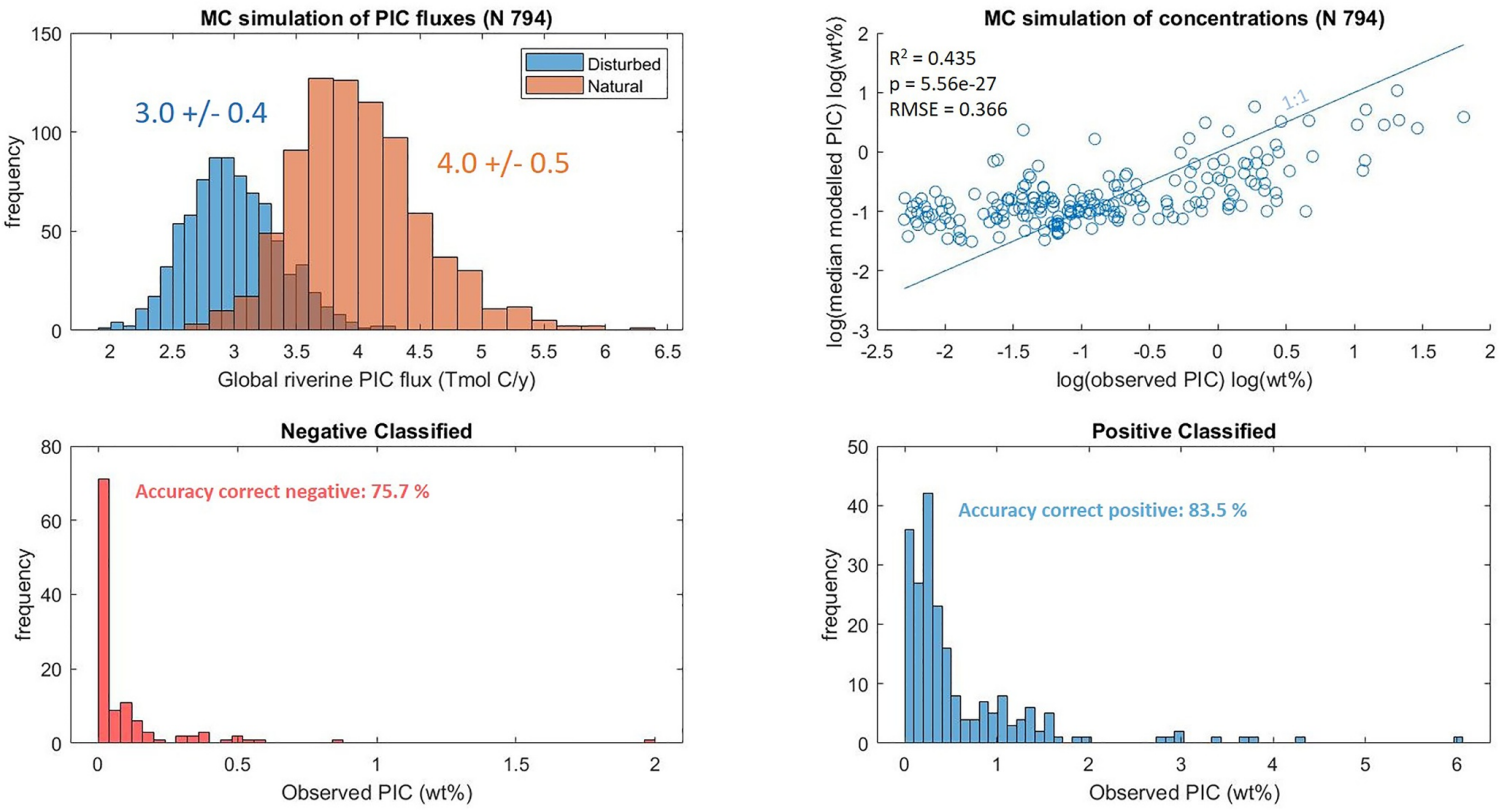
Results (upper) and performance (lower) of the Monte Carlo (MC)‐refined regression procedure. Histograms (upper left panel) show the distribution of natural and anthropogenically disturbed global Particulate inorganic carbon (PIC) fluxes in Tmol C/y (times 0.012011 yields Pg/y). The upper‐right panel assesses the performance of the quantitative prediction via SR (1:1 line = perfect prediction). The lower panels evidence the performance of an exemplary qualitative model (left: negative classifications (= No PIC present, correct predictions are <0.1 wt%); right: positive classifications (= PIC present, correct predictions are >0.1 wt%). N is the number of accepted MC simulations and RMSE is the root mean squared error.

We calculate that currently 3.1 ± 0.3 Tmol PIC are annually discharged to the coastal ocean. The prehuman flux was 4.1 ± 0.5 Tmol PIC/y (Figure 1, Table 2), accounting for damming and soil erosion (by the underlying WBMSed 2.0 model; Cohen et al., 2013, 2014). The 25% reduction is dominated by particle retention in reservoirs. The uncertainty of 10% appears low, considering the much larger uncertainty of sediment fluxes (50%), observed PIC concentrations (50%), and quantitatively modeled PIC concentrations (factor 4) (Supporting Information S1). The reason is the low uncertainty of PIC presence: correct negative classifications (= no PIC present) (75.7% accuracy) have a lower range of 0–0.1 wt%, and basins with less than 10% SC are assumed to have a PIC concentration and error of 0 wt%, reducing variability of results and errors. Positive classifications (= PIC present) are similarly accurate (83.5%). Moreover, modeled PIC concentrations are within a smaller range than observed values, that is, the model is biased. Very small values, that is, PIC <0.1 wt%, will not drastically affect results especially because the global flux is dominated by a few large rivers (see below). Miscalculations in PIC‐rich rivers could be more critical to the assessment, for example, the Rhone river is a comparably small river in terms of sediment discharge but a major contributor to the global PIC flux because of high PIC concentrations. However, for most of the important rivers, measurements are available, and thus this uncertainty is accounted for (Supporting Information S1). Therefore, the global flux and flux‐weighted average concentrations are rather robust. Notably, these uncertainties do not account for inaccuracies in the input data sets.

**Table 2:** Comparison of the Herein Presented Results and Literature‐Based Estimates of Global Average PIC Concentration (cPIC, Flux‐Weighted Mean, Median, and Mixture of Median and Mean, Respectively), Suspended Sediment Discharge (fTSS, Global Sum), and PIC Flux (fPIC, Global Sum)

Variable (unit)	cPIC (wt%)	cPIC (wt%)	cPIC (wt%)	fTSS river (Gt/y)	fPIC river, prehuman (Tmol C/y)	fPIC river, present day (Tmol C/y)	fPIC river, actual (Tmol C/y)	fPIC atmosphere (Tmol C/y)	fPIC cryosphere (Tmol C/y)
Value	**0.26**	0.42	0.7	16	**4.1**	**3.1**	10.4	**0.25**	**0.78**
Range	**0.24–0.28**	0.1–0.7	0.4–1	12–20	**3.6–4.6**	**2.8–3.4**	4.0–16.7	**0.10–0.40**	**0.48–1.12**
Reference	This study (fwm,model)	This study (med, obs.)	Literature (1–4)	Literature (5–8)	This study (model)	This study (model)	Literature (1–10)	Literature (11,12)	Literature (13–15)

*Note*. References: 1: Meybeck (1982), 2: Viers et al. (2009), 3: Savenko (2007), 4: Bayon et al. (2015), 5: Beusen et al. (2005), 6: Milliman and Farnsworth (2011), 7: Syvitski and Kettner (2011), 8: Cohen et al. (2014), 9: Middelburg et al. (2020) based on Canfield, (1997) and Beusen et al. (2005), 10: Meybeck (1993), 11: Journet et al. (2014), 12: Jickells et al. (2005), 13: Overeem et al. (2017), 14: Raiswell et al. (2008), 15: Wadham et al. (2013). med, median; fwm, flux‐weighted mean; obs, observations; wo, without. "Literature" indicates values and ranges that were calculated from published values ("first‐order" estimates, Supporting Information S1, gray columns). "This study" refers to values we derived in this contribution (2 Methods and Procedures, Supporting Information S1). Bold numbers indicate the values suggested for further use. Conversion from Tmol C/y to Pg/y by a factor 0.012011.

**Figure 2:**
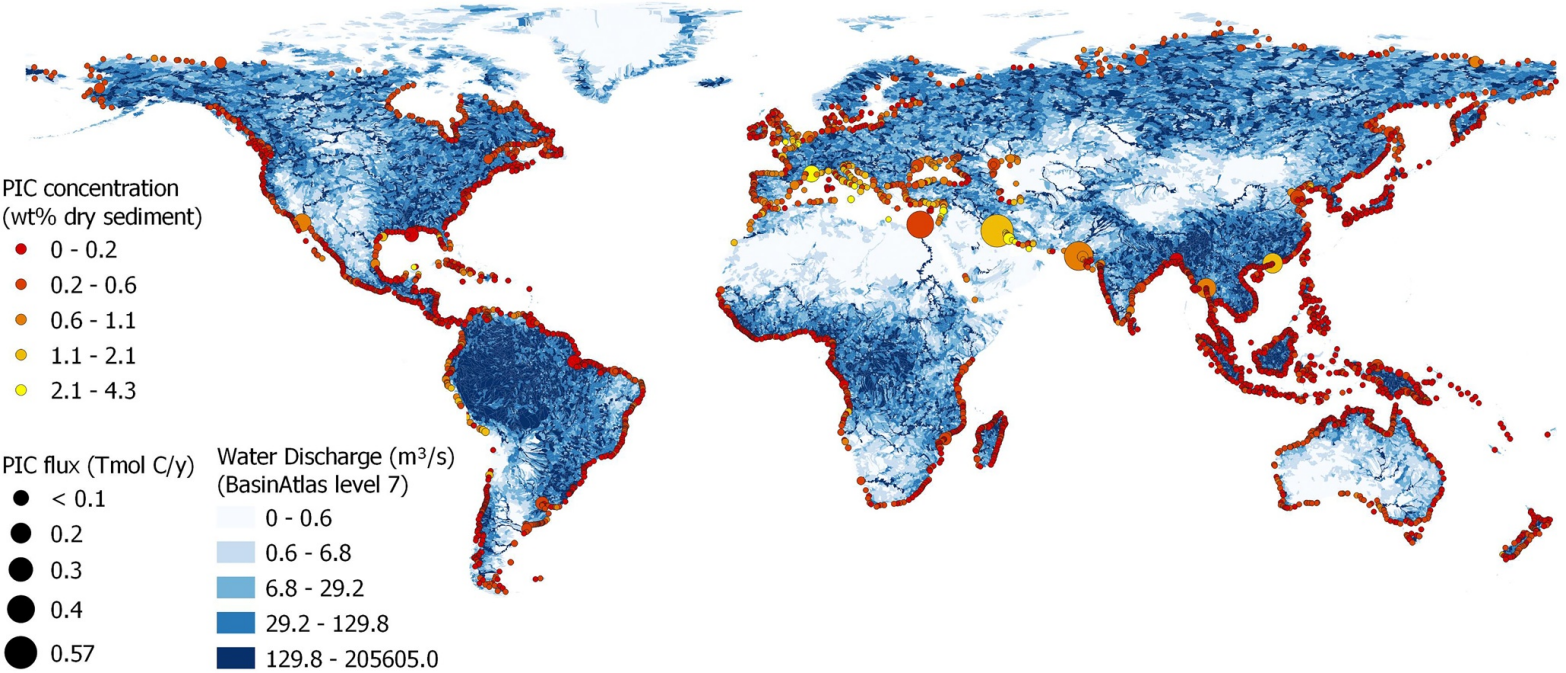
Map of the model results. Point data along the coast are the result of this study (mean of 794 accepted Monte Carlo simulations). Size scales with the magnitude of the Particulate inorganic carbon ((PIC) flux (Tmol C/y) based on prehuman sediment discharge and color is related to PIC concentration (wt %). For comparison, blue colors indicate natural annual mean water discharge (m^3^/s) (Linke et al., 2019). Conversion of fluxes to Pg/y by a factor 0.012011.

For instance, the global lithological map (GLiM) has an accuracy of only ∼60% compared to point observations (Hartmann & Moosdorf, 2012). The flux‐weighted mean PIC concentration of 0.26 ± 0.03 wt% is lower than the median of PIC‐bearing rivers only (0.41 ± 0.01 wt%, excluding PIC‐free rivers), implying ∼40% of the riverine sediment flux to be PIC‐free. Both are statistically indistinguishable from the median of observed basinal averages (0.35 ± 0.3 wt%), covering ∼35% of the global sediment flux (Cohen et al., 2014). Additionally, some authors used mean values, which are more susceptible to outliers caused by small rivers and are typically higher than medians (because of log‐normal distributions). High PIC concentrations are rarely found in rivers with high discharge, except for a few large rivers draining markedly dry (e.g., the Nile and Euphrates‐Tigris systems) and/or mountainous (e.g., the Indus system) catchments (Figure 2).

From a total of 3365 catchments considered, 862 basins contribute ∼99% of the total riverine PIC flux, while the 10 catchments with the largest PIC fluxes, situated in South‐East Asia, Arabia, Europe, and North America, already sum up to ∼53%. The Euphrates‐Tigris system (13.3%), the Indus (10.3%), and the Nile (8.9%) alone contribute 32.6% of the total global PIC flux, followed by Yangtze (4.5%), Salween (4.4%), Colorado (USA, 3.4%), Rhone (2.8%), Huanghe (2.1%), Mississippi (2.0%), and the Ganga‐Brahmaputra system (1.6%). About two‐thirds (2.7 Tmol PIC/y) are delivered to the coastal ocean between 15°N and 45°N, contrasting riverine DIC, OC, total solute, and bulk sediment fluxes (Hartmann et al., 2014; Ludwig et al., 1996; Milliman & Farnsworth, 2011). The anthropogenic reduction of the global PIC flux is dominated by the decreasing contribution of the Nile due to intense damming (∼8 of 25%).

The present PIC flux related to atmospheric dust deposition is 0.25 ± 0.15 Tmol C/y, which is ∼8% of the riverine PIC flux and ∼0.3% of the total riverine carbon flux (∼71 Tmol C/y, Supporting Information S1). Thus, the atmospheric contribution is negligible in global mass balances. PIC related to meltwater discharge and ice‐rafted debris from Greenland and Antarctica together contribute another 0.8 ± 0.3 Tmol PIC/y, which is ∼26% of the present‐day riverine PIC flux and ∼1% of the total river carbon flux (Supporting Information S1). In total, ∼4 Tmol of PIC arrive in the ocean annually (∼5 Tmol when considering natural river discharge, see Table 1)

## 5 Discussion

### 5.1 Natural Controls of PIC and Their Variation Through Time

**Figure 3:**
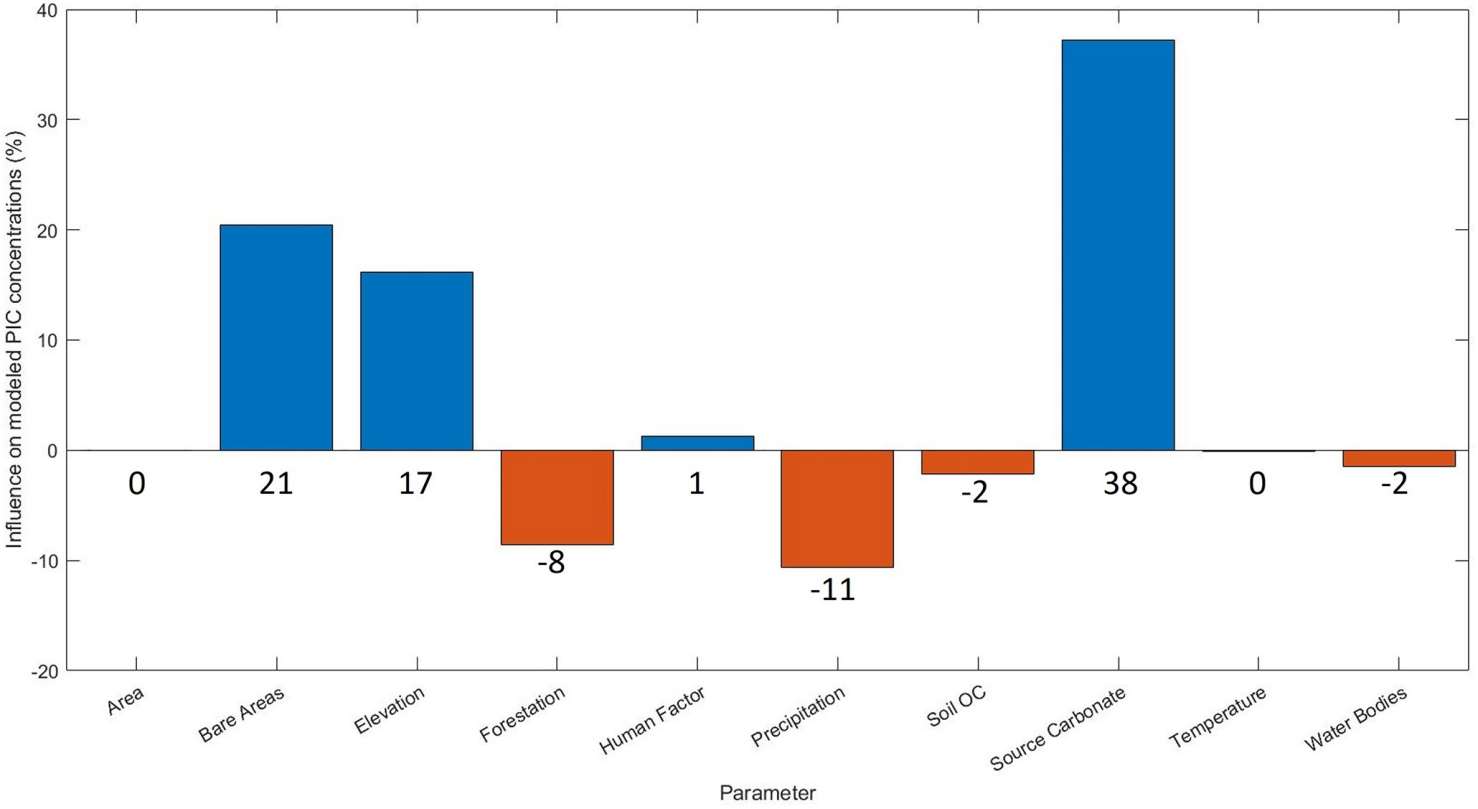
Relative importance of the different variables to our model results as assessed by the coefficient of determination (R^2^) between the variable in question and the median result of 794 high‐quality Monte Carlo simulations. The correlation coefficient gives the direction of influence (orange: negative, blue: positive). Individual values are indicated above the bars. OC: Organic Carbon. Variables as in Table 1.

The relevance of each variable to the model was assessed through the coefficients of correlation and of determination between the individual variable and the median model outcome, being independent of nonlinearity and multicollinearity (Figure 3). A strong positive influence of SC on PIC concentrations is eminent from these procedures (+38%, Figure 3). SC includes carbonate from soils (∼3% carbonate on average) and unconsolidated sediments (∼2%–4%) but is dominated by lithological (bedrock) contributions (∼20% on average). This is because terrestrial carbonate weathering is dissolution‐dominated, which arises from fast dissolution and high solubility (Lasaga, 1984; Morse & Arvidson, 2002). This contrasts with the precipitation‐dominated behavior of silicates, producing clay minerals and oxides characteristic to soil assemblages (Brantley et al., 2008; Lasaga, 1984; Ma et al., 2011; Morse & Arvidson, 2002).

Generally, a riverine suspended sediment is a mixture of source rocks, their solid weathering products (soil and sediment), organic matter, and material of anthropogenic origin with additional in‐stream processing. Thus, the differences between SC and PIC may arise from preferential dissolution of carbonates compared to silicates in the weathering zone (= soil) before erosion and also from in‐stream dissolution (Dornblaser & Striegl, 2009), precipitation (e.g., Kempe & Emeis, 1985; Négrel & Grosbois, 1999), and particle sorting during transport (e.g., Bouchez et al., 2011; Garzanti et al., 2011). According to our results, humans did not (yet) influence PIC concentration significantly on a global scale (influence of human factor is only 1%, Figure 3), while they severely reduced PIC fluxes through their impact on suspended sediment discharge (see Section 4.2 Human activities and riverine carbon). A thick soil cover can only develop if chemical weathering rates exceed material removal by erosion (Ferrier & West, 2017; West, 2012) and it is promoted by biological activity. Especially, forestation stabilizes the soil, disintegrates pristine rocks, and introduces organic acids and ligands, increasing mineral solubilities (Brantley et al., 2017; Calmels et al., 2014). This view is supported by the negative impact of variables in favor of soil formation and dissolution, such as precipitation (−11%) and forestation (−8%). In contrast, the organic carbon content of soils and temperature, which may influence dissolution kinetics, does not seem to play a major role for PIC concentrations, nor do catchment size or the extent of water bodies (relatable to the residence time of the particles within the fluvial system). The more prominent influence of (rock) erosion on PIC concentrations is evident from the large influence of related variables, namely, elevation (+17%) and the extent of bare areas (+21%). Rapidly eroding, mountainous terrains are characterized by fast, efficient transport and diminutive sediment storage (Hilton & West, 2020; Milliman & Syvitski, 1992), limiting both the extent of soil formation (Dixon and von Blanckenburg, 2012; Jenny, 1941) and in‐stream dissolution.

This interpretation that PIC concentrations increase with erosion is apparently inconsistent with increasing carbonate dissolution, following pyrite oxidation and sulfuric acid production upon accelerated erosion as observed in shale‐dominated terrains (Bufe et al., 2021; Calmels et al., 2007; Torres et al., 2014). However, we do not only consider shale‐rich, but all carbonate‐bearing (>10%) terrains in this analysis, which could obscure such relationships. Such an apparent inconsistency was noted for other global scale compilations as well (Bufe et al., 2021). Moreover, PIC is predominantly produced by the physical disintegration of pristine rocks and soils, while this same process promotes dissolution and oxidation kinetics. Thus, trends in PIC concentrations and carbonate dissolution do not strictly oppose each other but may even covary in rapidly eroding terrains.

**Figure 4:**
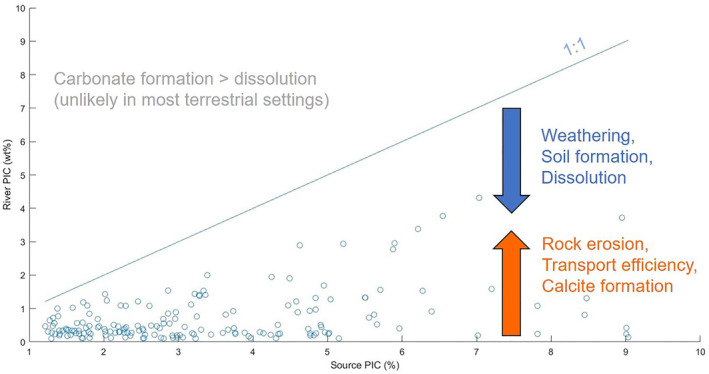
Relationship of river Particulate inorganic carbon (PIC) and source PIC. Source PIC (12% of SC) includes sediment and soil contributions but is dominated by rocks. In‐stream dissolution and contributions of weathered material decrease river PIC, while rock erosion has a pronounced positive effect by contributing source rock. Transport efficiency and in‐stream precipitation can further enhance PIC concentrations at the river mouth. The unit of Source PIC (%) is wt% PIC in the given percentage of carbonate within the upstream outcrop area.

In summary, the rather slowly changing (10^3^–10^5^ y) tectonic, physiographic, and lithological settings seem to exert a dominant control on PIC concentrations as demonstrated by the eminent role of SC and elevation in our model. Superimposed on this base‐line situation, much faster variations in climatic and vegetation patterns seem to affect the relative contributions of weathered, PIC‐poor soil and pristine, PIC‐rich source rock (Figure 4).

Although heavily discussed (Caves Rugenstein et al., 2019; Foster & Vance, 2006; Willenbring & Von Blanckenburg, 2010), many observations suggest that soil formation and/or chemical weathering decreased during cold, dry periods (Berner et al., 1983; Dixon et al., 2016; Jenny, 1941; Schachtman et al., 2019), potentially increasing the ratio of pristine source rock to weathered soil in suspended sediments and thus the riverine PIC flux. Moreover, glacial activity during cooler periods may accelerate erosion and thus PIC production and potentially preservation (because of decreased residence times). This is supported by an increase in the detrital carbonate fraction in glacial sediments of the Gulf of Lyon compared to sediments deposited during interglacial periods (Pasquier et al., 2019). An indication of increased cryogenic PIC deposition in response to ice‐sheet dynamics is provided by the so‐called "Heinrich events," which are unusual accumulations of coarse carbonate‐rich detritus in marine sediment (Bond & Lotti, 1995; White et al., 2016). In contrast, thawing permafrost exposes old but fresh organic matter that is rapidly respired (e.g., Walz et al., 2017), potentially increasing PIC dissolution (Aller, 1982; Archer et al., 1989; Calmels et al., 2014; Oelkers et al., 2011; Zolkos et al., 2018). Consistently, although not solely related to carbonate weathering, an increased riverine export of DIC in response to the recent warming was reported from large Arctic rivers (Drake et al., 2018; Zolkos et al., 2020).

However, recent observations and theories challenge this simple view (Caves Rugenstein et al., 2019; Foster & Vance, 2006; Willenbring & Von Blanckenburg, 2010), implying more complex and transitional spatiotemporal dynamics of erosion (Chen et al., 2018; Foreman et al., 2012; van de Schootbrugge et al., 2020) and carbonate weathering (Gaillardet et al., 2019; Zeng et al., 2019) and, consequently, of riverine inorganic carbon export. Additionally, environmental conditions and, consequently, carbonate dissolution in the (coastal) ocean are expected to change over multiple time scales, ranging from seasons and decades (Cai et al., 2011; Wallace et al., 2014) to geological time scales (Broecker, 1982; Ganeshram et al., 2000; Sluijs et al., 2013) with implications for the magnitude and timing of contribution of PIC to oceanic inventories.

### 5.2 Human Activities and Riverine Carbon

Rivers annually deliver about 31.5 Tmol DIC, 19.1 Tmol DOC, and 17.4 Tmol POC to the ocean (Table 3). Including 3.1 Tmol PIC/y increases the total riverine carbon export (TC) to 71.1 Tmol C/y, equating a contribution of 4%, which is within the uncertainty of the estimates excluding PIC (Table 3). An accurate and precise knowledge of the riverine carbon export is necessary to understand the distribution and fate of anthropogenic carbon perturbations (Friedlingstein et al., 2020; Resplandy et al., 2018). Over the past century, these riverine carbon fluxes have changed and continue doing so, likely in response to climate change and local human activities, such as industrialization, changes in land use, hydrology, and agricultural practices (Drake et al., 2018; Lambert et al., 2017; Li et al., 2019; Liu et al., 2020; Noacco et al., 2017; Raymond & Hamilton, 2018; van Hoek et al., 2021; Zeng et al., 2019).

**Table 3:** Summary of the Riverine Carbon Export (in Tmol C/y)

	DIC	PIC	DOC	POC	TC
Modern global river export (Tmol C/y)	31.5	3.1	19.1	17.4	71.1
Percentage of TC	44.3	4.4	26.9	24.5	100
Range (Tmol C/y)	26.6–36.3	2.8–3.4	14.2–30.0	14.2–20.0	57.8–89.7
Human disturbance (%)	+13.5*	−24^S^	−13^D^	−13^D^	−2
Range (%)	+9.8 – +17.1	−5.6 – −39.0	−12.8– −13.2	−12.8 – −13.2	−4 to + 0.7
Source	Literature	This study	Literature	Literature	This study and Literature.

*Note.* DIC: (Amiotte Suchet et al. (2003); Gaillardet et al. (1999); Hartmann et al. (2014); Li et al. (2017); Ludwig et al. (1996, 1998); Meybeck (1982), DOC: (Aitkenhead and McDowell (2000); Dai et al. (2012); Harrison et al. (2005); Li et al. (2019); Ludwig et al. (1996, 1998), POC: Beusen et al. (2005); Galy et al. (2015); Li et al. (2017); Ludwig et al. (1996, 1998); Meybeck (1982), Superscripts: ^S^: By changes in sediment flux only, ^D^: By damming only (Maavara et al., 2017; This study); *: By climate change and land‐use change for carbonate weathering only (Zeng et al., 2019). Human disturbance of TC is the bulk effect as indicated for DIC, PIC, DOC, and POC. “Literature” indicates averages and ranges taken from the abovementioned studies (gray columns). TC represents the sum of our PIC estimate and DIC, DOC, and POC estimates from literature. Conversion to Pg/y by a factor 0.012011.

The net effect of human activity on riverine sediment discharge is a ∼10% reduction, dominated by damming (Cohen et al., 2014; Syvitski et al., 2005), resulting in an even higher reduction of riverine PIC (∼24%, Table 3) and OC fluxes (∼13%, Maavara et al. (2017)). The differences between those fractions are related to the non‐even spatial patterns of riverine carbon and sediment discharge (Ludwig et al., 1996; Milliman & Farnsworth, 2011, Figure 2). Damming also increases the residence time of particles in the riverine realm (Rueda et al., 2006), where PIC and POC are commonly remobilized by dissolution/degradation. However, organic matter degradation and burial in reservoirs are very heterogeneous and dependent on reservoir ages (Maavara et al., 2017). Low importance for the model (−2%) for the extent water bodies, including reservoirs, indicates a rather negligible effect of reservoirs on PIC concentrations on the global scale. Our human factor is not an important predictor in the model (1%), despite the expected influence of lime fertilizers (Haynes & Naidu, 1998; Shoghi Kalkhoran et al., 2019; Zeng et al., 2019), of cement (Horvath, 2004) and of human‐induced soil erosion, the latter affecting the active weathering zone (Govers et al., 2014). PIC could also be reduced by increasing dissolution through industrial or agricultural acids (Perrin et al., 2008; Webb & Sasowsky, 1994; Wicks & Groves, 1993). Eventually, the lack of resolution between those positive and negative influences in our human factor obscures a clearer relationship. Thus, more detailed studies on the different human influences on riverine carbonate are required. Notably, the human influence is correlated with the observed PIC concentrations, but this spurious relationship stems from collinearity of SC and human population, both being high in Southeast Asia, Europe, and North America, confirming our method is correcting for multicollinearity.

A 24% reduction of PIC fluxes equates to only 1.4% of the total riverine carbon flux (TC). The damming‐related decrease in organic carbon fluxes (13% of OC, Maavara et al. (2017)) results in another 6% reduction of TC. In contrast, carbonate dissolution‐related DIC fluxes likely increase(d) by ∼13.5% in the period 1950–2100 as a consequence of climate change and land‐use change (Zeng et al., 2019). Such an increase in DIC fluxes would result in a 5.6% increase in TC, partially compensating the reduction of OC and PIC in terms of total carbon export (total disturbance: −2%). This is consistent with the estimation of a somewhat stable riverine TC export as a result of in‐stream removal of anthropogenic carbon by POC deposition and respiration (Cole et al., 2007; Regnier et al., 2013; van Hoek et al., 2021).

The bulk anthropogenic effect on total global riverine DIC fluxes remains elusive (Raymond & Hamilton, 2018) and human activities other than dam‐building impact terrestrial and freshwater carbon cycling (van Hoek et al., 2021). Notably, humans also change conditions at the site of riverine PIC deposition: The current coastal ocean acidification in response to anthropogenic emissions and eutrophication (Borges & Gypens, 2010; Carstensen & Duarte, 2019) could enhance PIC dissolution, acting as a heterogeneous buffer (Middelburg et al., 2020).

### 5.3 Implications for Oceanic Mass Balances

The fate of the detrital carbonate flux in the marine realm, that is, PIC burial or dissolution, determines the implication of the global PIC flux for oceanic mass balances (Middelburg et al., 2020). PIC preservation may affect global estimates of marine carbonate burial, while PIC dissolution would translate to an additional input of Ca, Mg, Sr, C, and alkalinity to the marine solute inventories. Because the scientific community lacks a reliable global quantification of these aspects, we discuss the following questions

1. Where is river PIC deposited?
2. Does PIC deposition influence global estimates of carbonate burial?
3. Does PIC dissolve and alter oceanic mass balances of Ca, Mg, C, Sr, and alkalinity?

#### 5.3.1 Where Is River PIC Deposited?

On time scales of years to centuries, a major fraction of the riverine suspended matter remains in the estuary (often ∼40%–60%), while the rest is deposited along the shelves and continental slopes with little escape toward the deep sea (Dyer, 1995; Meade, 1972; Wright & Nittrouer, 1995). However, sediment dynamics in river‐dominated ocean margins are highly variable in space and time, including deposition near the river mouth and subsequent lateral advection to more calm environments as well as transport toward the slope (Geyer et al., 2004; McKee et al., 2004). Saderne et al. (2019) emphasize that some coastal ecosystems, such as mangrove forests and seagrass meadows, efficiently trap and dissolve such detrital carbonate from external sources.

Global sea level fall under cooler climates (average Pleistocene state) exposes the PIC‐rich shelf to erosion, shifting depocenters to the slope, where PIC may be further transported to and/or dissolved in the open ocean (Filippelli et al., 2007; Kump & Alley, 1994; Tsandev et al., 2010). Thus, on larger time scales (>10^3^ years), most of the riverine PIC that do not dissolve on a short time scale (1–10^2^ years) will be transported to the slope. A significant fraction may, however, have dissolved before remobilization or remain at the initial site of deposition (preservation of the former estuary/shelf).

#### 5.3.2 Does PIC Deposition Influence Global Estimates of Carbonate Burial?

Riverine PIC burial on the shelves may be implicitly included in carbonate mass accumulation (CMA) rate estimates derived from carbonate content, density, and sediment accumulation rates, although microscopic criteria were established to distinguish biogenic and detrital carbonates (Milliman, 1974). However, hot spots of carbonate burial do generally not coincide very well with hot spots of riverine suspended sediment deposition (i.e., carbonate‐poor shelves) (O’Mara & Dunne, 2019). Moreover, the dissolving PIC fraction and the fraction that remains at the initial site of deposition (i.e., is not re‐eroded from the former estuary over longer time scales) do not contribute to estimates of carbonate burial on the slope. Therefore, we believe that the effect of riverine PIC deposition on CMA‐derived estimates of biogenic carbonate burial is rather limited. In contrast, carbonate burial estimates derived from mass balances (e.g., van der Ploeg et al., 2019) or solution chemistry (e.g., Chung et al., 2003) are directly affected by PIC dissolution.

#### 5.3.3 Does PIC Dissolve and Alter Oceanic Mass Balances of Ca, Mg, C, Sr, and Alkalinity?

Marine surface waters are supersaturated with respect to most carbonate minerals (Milliman, 1974; Peterson, 1966). Therefore, provision of carbonate mineral surfaces by PIC discharge, energetically favoring nucleation of these same minerals, may trigger inorganic carbonate precipitation in the water column (Wurgaft et al., 2016). TIC/TOC ratios of sediments from the Huanghe estuary, China (Gu et al., 2009; Yu et al., 2018), and trends in alkalinity/DIC ratios in the marginal Red Sea support this view (Wurgaft et al., 2016). Compared to marine carbonate compensation, PIC will rapidly settle in the shallow coastal ocean, and the degree of carbonate saturation varies with depth and across different local environments at the seafloor (Aller, 1982; Boudreau & Canfield, 1993). As chemical conditions, especially pH, vary within the sediment column, carbonate may even be dissolved in the upper parts of the sediment column but formed in the lower, more alkaline parts (Aller, 1994).

Carbonate dissolution at the sediment‐water interface and in diagenetic settings is well known (Aller, 1982; Archer et al., 1989; Sulpis et al., 2017). In these settings, aerobic degradation of organic matter may drive carbonate dissolution via the production of CO_2_ and other acidic compounds (Aller, 1982; Oelkers et al., 2011). Anaerobic degradation produces reduced metabolites, such as ammonium, sulfide, and iron (II), most of which form strong acids upon upward migration and subsequent reoxidation in the bioturbated zone, which drastically reduces carbonate saturation (Aller, 1994; Boudreau & Canfield, 1993) and may alter the carbon cycle coupling of subsequent dissolution (Beaulieu et al., 2011; Huang et al., 2017; Liu et al., 2018; Torres et al., 2017).

Carbonate dissolution may also be influenced by biological activities, such as seagrass root oxygen loss, sponge boring, and bioturbation (Burdige et al., 2008; Mackenzie & Andersson, 2011; Saderne et al., 2019). Substantial riverine PIC dissolution was observed in the maximum turbidity zone of the eutrophic Loire estuary, France (Abril et al., 2003). Moreover (detrital) carbonate dissolution driven by eutrophication‐related bottom water acidification was observed in the Gulf of St. Lawrence, Canada (Nesbitt & Mucci, 2021) and in the Chesapeake Bay, USA (Shen et al., 2019). The proposed total flux of ∼5 Tmol PIC/y corresponds to ∼10 Tmol (eq)/a of alkalinity (∼30% of dissolved equivalent), which is ∼30% lower than the estimate of Middelburg et al. (2020). Despite integration of groundwater discharge, marine organic matter burial, anaerobic processes, and marine silicate weathering, the modern ocean alkalinity budget is marked by an imbalance of ∼25% of the output by carbonate burial (59 Tmol (eq)/y in the coastal and open ocean). Half of this imbalance can be closed by the inclusion of riverine PIC fluxes, assuming that terrestrial PIC will either dissolve or bias the estimates of carbonate burial (Graphical Abstract). Part of the residual imbalance could be attributed to other diagenetic processes in the coastal zone, such as marine alumo‐silicate weathering (Gislason et al., 2006; Hong et al., 2020; Jones et al., 2012; Torres et al., 2020) and carbonate diagenesis (DePaolo, 2004; Fantle et al., 2010; Paytan et al., 2021). However, part of the imbalance could be real, considering that the residence time of carbonate ions in the ocean (∼100 ky) is larger than the time since the last glaciation (Middelburg et al., 2020; Milliman, 1993).

The inputs of ∼5 Tmol PIC/y (rivers + cryosphere) further imply ∼4.75 Tmol Ca/y, ∼0.25 Tmol Mg/y, and ∼5 Gmol Sr/y, assuming ideal stoichiometry, 10% dolomite (typical value in *GloRiSe* v1.1), and 1,000 ppm Sr in calcite and dolomite. This equates to ∼34.7% (Ca), ∼4.6% (Mg), and ∼8.9% (Sr) of the respective dissolved equivalents, representing the current major input terms of the respective marine mass balances (Berner & Berner, 2012; Krabbenhöft et al., 2010; Mayfield et al., 2021; Tipper et al., 2006, 2010). As dolomites typically exhibit much lower dissolution rates than calcites (Pokrovsky et al., 2005), Mg addition by PIC dissolution is probably even smaller and thus negligible. So far, none of these highly discussed budgets could be consensually balanced neither at the present state nor in reconstructions of the past—a conundrum persisting already for decades (Berner and Berner, 1987, 2012; Hong et al., 2020; Jones et al., 2012; Krabbenhöft et al., 2010; Mayfield et al., 2021; Middelburg et al., 2020; Milliman, 1993; Shalev et al., 2019; Tipper et al., 2006, 2010).

**Figure 5:**
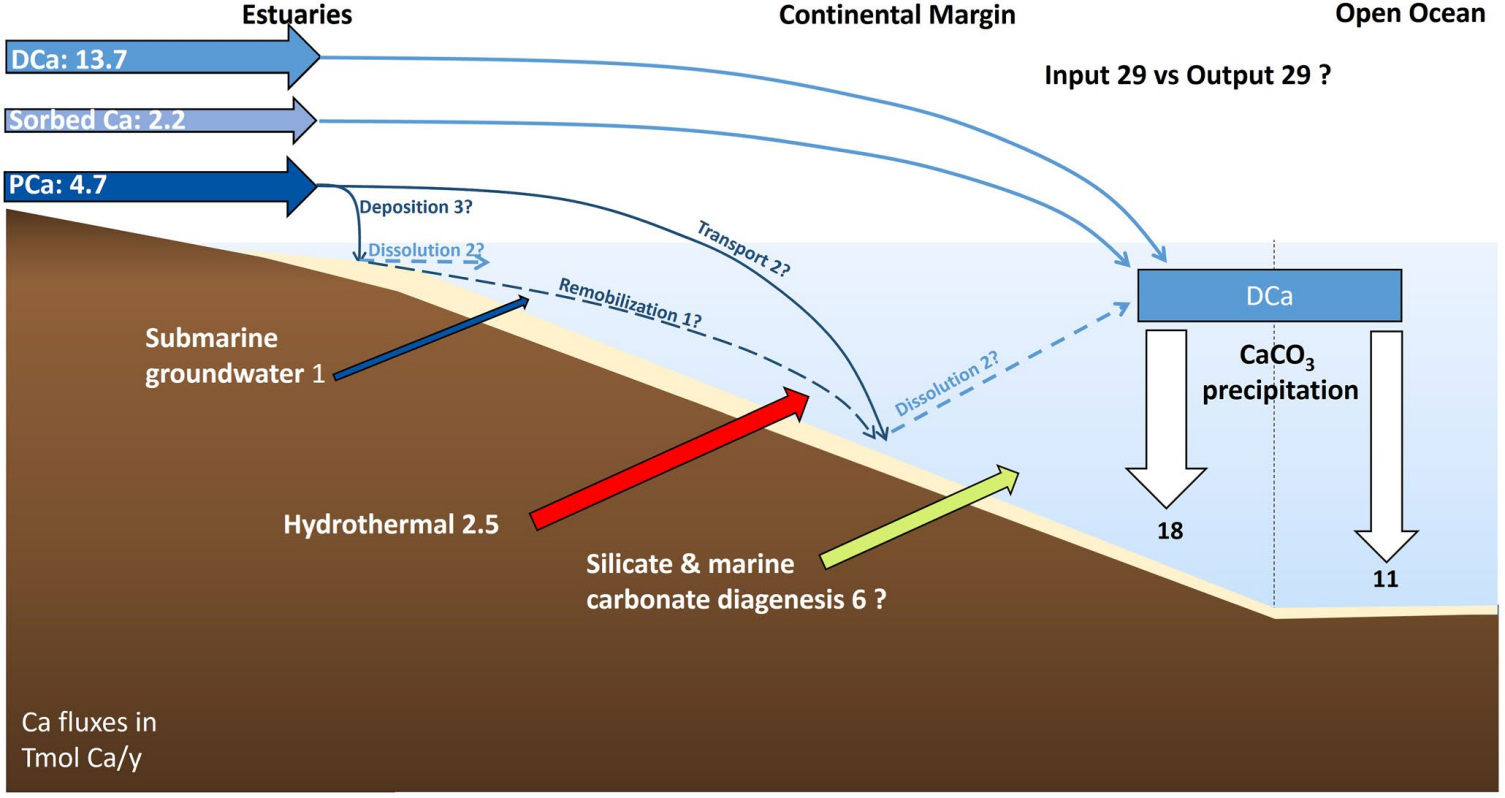
Illustration of the modern ocean calcium budget and how it may be complemented by the inclusion of the riverine PCa flux. The fate of PCa (particulate Ca) in the ocean is, however, uncertain (details in the main text). Fluxes are given in Tmol Ca/y. CaCO_3_ burial fluxes are from Middelburg et al. (2020), Milliman (1993), and O’Mara and Dunne (2019). River‐sorbed Ca is from Müller et al. (2021a), Müller et al. (2021b), DCa (dissolved Calcium) and groundwater inputs are from Mayfield et al. (2021), and the hydrothermal flux and marine carbonate diagenesis (3–4 Tmol Ca/yr) are from DePaolo (2004). The silicate diagenesis flux of Ca is assumed to fill the residual imbalance of ∼2 Tmol Ca/yr.

Riverine PIC input also impacts the Ca cycle (Figure 5). Apart from riverine dissolved Ca fluxes, submarine groundwater discharge (1 Tmol Ca/yr, Mayfield et al., 2021) and hydrothermal processes (2–3 Tmol Ca/yr, DePaolo, 2004) were invoked to balance the high‐output fluxes by carbonate burial but still leave an imbalance of 36% that can be reduced further by 16% through consideration of PIC fluxes. The remaining 20% could be attributed to submarine weathering of volcanogenic silicate debris (Gislason et al., 2006; Hong et al., 2020; Jones et al., 2012; Torres et al., 2020) and/or carbonate diagenesis (3–5 Tmol Ca/yr, DePaolo, 2004; Fantle et al., 2010). However, carbonate precipitation in early diagenetic settings currently represents an additional sink of ∼1 Tmol Ca/yr and ∼2 Tmol (eq)/y of alkalinity and is related to anaerobic oxidation of organic matter and silicate weathering (Schrag, 2013; Sun & Turchyn, 2014; Torres et al., 2020). This flux is implicitly included into the mass balance of alkalinity (Graphical Abstract) by reducing the alkalinity source of the diagenetic reflux through carbonate dissolution (from DePaolo, 2004).

The fraction of detrital carbonates in coastal margin sediments was estimated to <10 and 50% in the Bengal fan (France‐Lanord et al., 2018) and in the Gulf of Lyon (Pasquier et al., 2019), respectively. If the long‐term biogenic carbonate burial on the slope is ∼2 Tmol C/y (Milliman, 1993) and all riverine PIC (natural: 4.1 Tmol C/y) either dissolves or is transported to the slope on long time scales, then 2.1–3.9 Tmol PIC/y (51%–95%) would need to dissolve in order to match these detrital fractions. This back‐of‐the‐envelope calculation is not a valid quantification of the globally dissolving PIC fraction but illustrates that PIC dissolution may indeed be significant. However, as argued above, the coastal ocean is a heterogeneous region with locally and temporally variable conditions, supporting carbonate dissolution, preservation or precipitation. Importantly, recrystallization still leads to an exchange with the marine element and isotope inventories (e.g., Fantle et al., 2010; Kastner, 1999; Paytan et al., 2021). A global estimation of the dissolving PIC fraction should account of this spatiotemporal variability and complex interactions of organic and inorganic particles within coastal sediments. Therefore, consideration of the detrital mineral flux of rivers to the ocean and its isotopic composition may help to solve long‐standing debates about imbalances in global biogeochemical cycles.

## 6 Next Steps

Improving our understanding of the role of PIC in global biogeochemical cycles involves a qualitative understanding and quantitative estimation of its fate in the marine realm as discussed in the previous section. This might be accomplished by tracing detrital components through isotopic approaches (e.g., France‐Lanord et al., 2018; Pasquier et al., 2019) and by diagenetic modeling of riverine particles involving all the important biogeochemical processes driving carbonate precipitation and dissolution (Meister et al., 2022; Torres et al., 2020).

**Figure 6:**
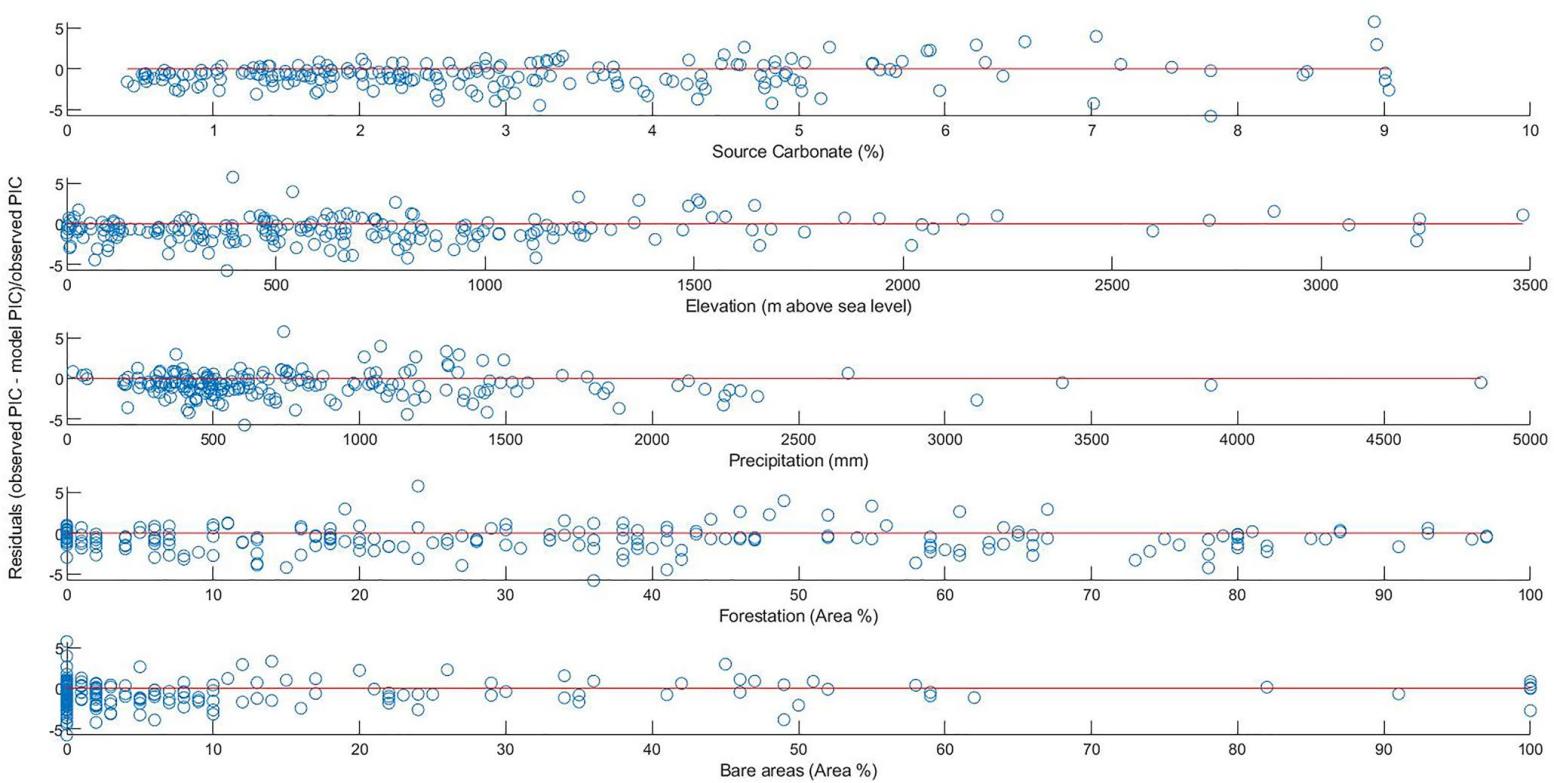
Analysis of distribution of relative residuals (fractional deviation from observations) among the most important predictors in the model.

Apart from this, the accuracy of the modeled riverine PIC concentration needs to be improved. As seen from Figure 3, the “SC” is the most important predictor used in the model, but it is also the least well constrained, critically increasing the relative misfit at high concentrations and downward biasing at low concentrations (Figure 6) This might be improved by a more thorough assignment of the carbonate content to the different lithological and unconsolidated sediment units through the integration of chemical or mineralogical analysis into the corresponding maps. However, limited precision of low concentration measurements might pose an analytical limitation. In highly forested regions, PIC concentrations seem to be systematically underestimated, which might be related to the type of forest. There is no obvious correlation of residuals to other important predictors (elevation, precipitation, and bare areas), suggesting that these variables are reasonably well captured. However, as discussed in Section 4.2 (Human activities and riverine carbon), the human factor used in this study is rather vague (Table 1) and might mask certain opposing effects, such as liming or locally increased dissolution by acid introduction. Therefore, a detailed analysis of these factors would be warranted.

Another important feature may be the accuracy and precision of measured PIC concentrations as most studies on sediment composition do not involve PIC analysis or at least do not report the carbonate content that was removed during sample preparation of organic carbon analysis (Müller et al., 2021a). A greater spatial and temporal (time series) coverage of such observations, especially in remote regions, such as Greenland and Antarctica, would assist in a more accurate upscaling. Similarly, vertically integrated sediment flux measurements, including estimates of bed load transport, could improve the quantification of not only PIC, but all river sediment‐related biogeochemical fluxes (e.g., Galy & France‐Lanord, 2001). Finally, contributions of Arctic continental ice‐rafting and from coastal erosion to PIC fluxes could be significant and lack any reliable estimate.

## 7 Conclusion

The riverine flux of PIC, that is, discharge of detrital carbonate minerals, represents a significant, yet mostly unaccounted chemical mass transfer in the Earth system (3.1 ± 0.3 Tmol C/y), currently contributing ∼4.4% to the total riverine carbon export. The prehuman flux was 4.1 ± 0.5 Tmol PIC/y; the 24% reduction is caused by particle retention in reservoirs, especially of the Nile River. Considering perturbations of riverine particulate and dissolved, inorganic, and organic carbon species in concert, the riverine export flux seems to have remained rather stable, while carbon speciation changed.

Although the fate of PIC in the ocean remains quantitatively unknown, oceanic element and isotope inventories of Ca and alkalinity are most probably affected by detrital carbonate dissolution in the coastal ocean with implications for conclusions deduced from their highly debated, but frequently used mass balances. PIC contributions to the oceanic budgets of Sr and total C are less important and Mg fluxes are insignificant.

Naturally, the concentration of PIC is controlled by catchment topography and surface lithology, that is, slowly changing tectonic factors (10^3^–10^5^ y scales) but also by climate and vegetation, which are subjected to much faster spatiotemporal variations. Similarly, marine conditions change through time so that related PIC dissolution may also vary. An additional, significant amount of detrital carbonate (0.8 ± 0.3 Tmol C/y) is exported from Greenland and Antarctica and responds to ice‐sheet dynamics, while eolian contributions can be neglected (at the present). These results imply a response of the global PIC flux to human activity and to natural changes in environmental and climatic conditions but also to the tectonic evolution of our planet.

## Conflict of Interest

The authors declare no conflicts of interest relevant to this study.

## Supporting information

Supporting Information S1

Data Set S1

## Data Availability

All data and scripts used for this study, along with a detailed manual and the Supporting Information S1, can be accessed via https://doi.org/10.5281/zenodo.6125880 (DOI: 10.5281/zenodo.6125880). Data from which figures were generated can also be found as Supporting Information S1 alongside the online version of this article.
