# Supplementary material for: Detrital Carbonate Minerals in Earth's Element Cycles
Source: Global Biogeochem Cycles. 2022 May 17;36(5):e2021GB007231. doi: 10.1029/2021GB007231 (PMC9285522; doi:10.1029/2021GB007231)
Supplement: Supplementary file 1 — Supporting Information S1 [file GBC-36-0-s002.docx]

Supplementary Information to
Detrital carbonate minerals in Earth’s element cycles

**Gerrit Müller*^,a^, Janine Börker^b^, Appy Sluijs ^a^, Jack J. Middelburg ^a^**

**^a^** *Department of Earth Sciences, Utrecht University, The Netherlands*

**^b^** *Institute for Geology, CEN (Center for Earth System Research and Sustainability), Universität Hamburg, Germany*

[*g.muller@uu.nl*](mailto:g.muller@uu.nl)

[*janine.boerker@uni-hamburg.de*](mailto:janine.boerker@uni-hamburg.de)

[*A.Sluijs@uu.nl*](mailto:A.Sluijs@uu.nl)

[*J.B.M.Middelburg@uu.nl*](mailto:J.B.M.Middelburg@uu.nl)

** corresponding author: Gerrit Müller
Mail:* [*g.muller@uu.nl*](mailto:g.muller@uu.nl)
Address: Room 318, Vening Meineszgebouw A, Princetonlaan 8a, 3584 CB Utrecht

## S1 Data compilation

To derive a model for the prediction of carbonate content in riverine suspended sediment from catchment properties, we compiled a dataset of riverine annual average PIC concentrations (*GloRiSe* v 1.1, Müller et al., 2021a) and respective catchment information, including the composition of rocks (*GLiM*, Hartmann and Moosdorf, 2012), unconsolidated sediment (*GUM*, Börker et al., 2018) and soil (WISE, Batjes, 2012), as well as a variety of hydro-environmental, topographical and land cover-related variables (*HydroBasins* v. 1.0, Linke et al., 2019). Annual median PIC concentrations were calculated separately for single observations, seasonal averages and annual averages from suspended and bed sediment samples with grain sizes < 125 µm (fine sand) for each location, following the calculation scheme of Müller et al., (2021b). In addition to direct PIC measurements (e.g. barometric or by Carbon analyzer), mineralogical and petrographic observations (Light Microscopy, X-Ray Diffractometry, Electron Microscopy or Raman Spectroscopy) were stoichiometrically converted to PIC:

$PIC [wt\%]=calcite*0.12+dolomite*0.125 \approx bulk carbonate*0.12 \approx limestone*0.12$ *(S1)*

The value 0.12 is the stoichiometric mass fraction of C in CaCO_3_ and 0.125 the one of C in CaMg(CO_3_)_2_. For samples, where no such information about their carbonate content was given, we used an empirical linear model based on CaO, MgO, K_2_O and Al_2_O_3_ to estimate PIC concentrations (Figure S 1; Table S 1).


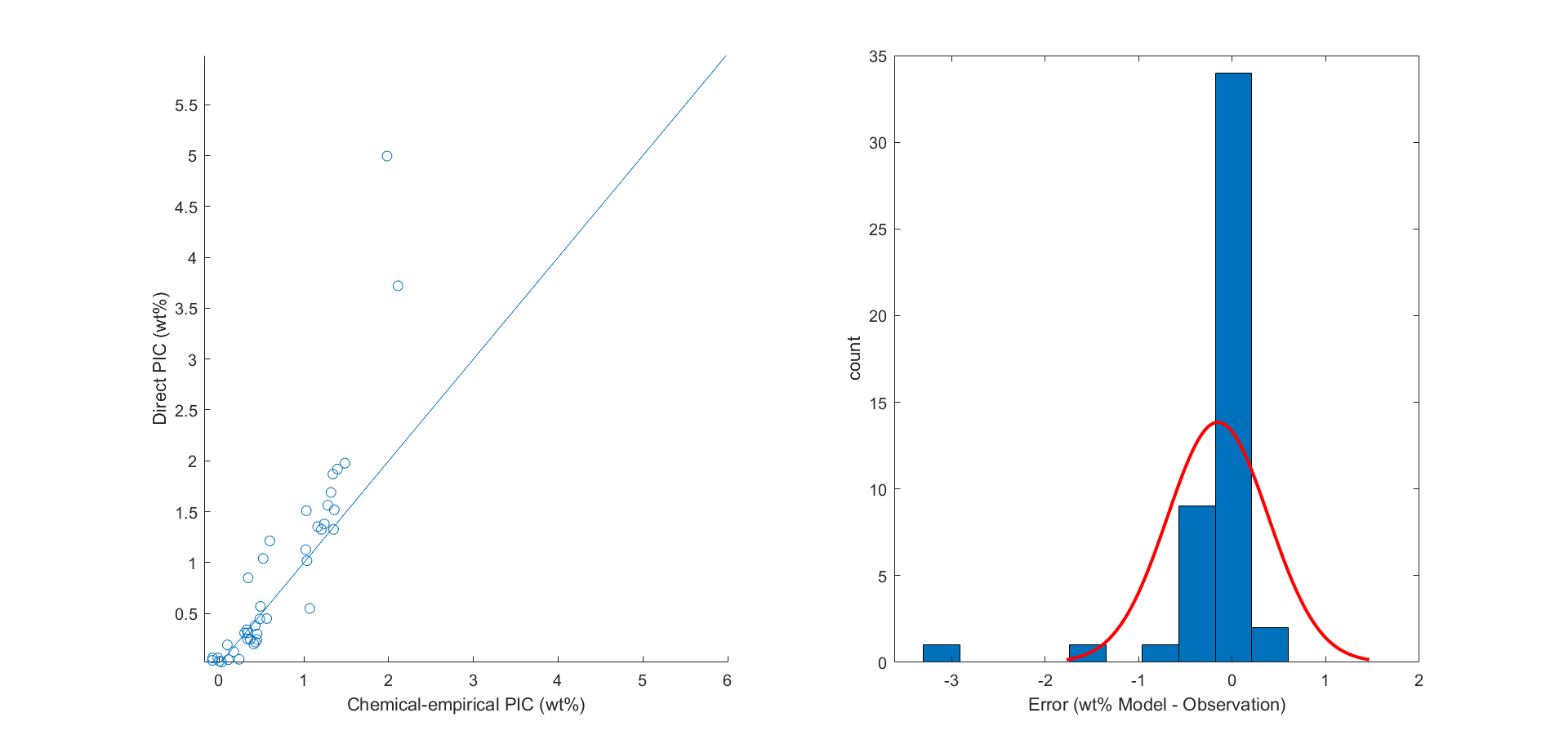


***Figure S 1*** *Performance of the mean coefficient, major-element based empirical model for the prediction of PIC from CaO, MgO, K_2_O and Al_2_O_3_ on the complete set of annual median PIC values from direct measurements (N = 49). The red line represents a fit of a Gaussian error distribution. The blue line represents the 1:1 line, i.e., a perfect prediction.*

The parameters of this relationship were derived by a linear regression of annual values from *GloRiSe* locations, for which major element composition was given along with direct PIC measurements, using a Levenberg-Marquardt algorithm. This empirical model was preferred over stoichiometric conversion of CaO to PIC and unmixing models, because it mitigates complications through sorbed, silicate-bound and phosphate-related CaO, difficulties associated with variable fractions of dolomite and ambiguity of the relationship between chemical and mineral composition. The model potentially overpredicts PIC in basalt-dominated catchments, as mafic mineral assemblages contribute elevated silicate-bound CaO and MgO contents not covered in the calibration. Therefore, we set locations manually to zero, at which the sum of basaltic and intermediate volcanics and plutonics and pyroclastics exceed the potential source carbonate content of the catchment (whose derivation is described below). Samples from locations on basaltic islands (e.g., Iceland), were excluded from the analysis. To estimate the precision of this model on the basis of uncertainty through limited sample availability and (potentially) insufficient coverage, the linear regression was repeated 20,000 times with different (80%) subsets selected by Latin hypercube sampling (LHS). LHS assures an even distribution of each (pseudo-)random subset over the whole range. The size subsets was chosen as a compromise retaining representativity and sufficient size and sub-set diversity. The optimal number of runs was chosen as the point where mean value and standard deviation of parameter estimates remained stable within < 0.01. Resulting parameters and uncertainties are given in Tab. S1 and yield an R^2^ of 0.81 (p < 0.01), when applied to the full original dataset. By comparing single measurements to flux-weighted mean values at ten locations with > 10 samples with sediment flux information available in *GloRiSe*, we estimate the mean relative uncertainty of the annual average PIC concentration to 50 % (median deviation 47.4 %, mean deviation: 54.9 %).

**Table S 1** Mean and standard deviation (SD) of 20,000 parameter estimates for the prediction of PIC from CaO, MgO, K_2_O and Al_2_O_3_.

|  | **CaO** | **MgO** | **K_2_O** | **Al_2_O_3_** |
| --- | --- | --- | --- | --- |
| **Mean Coefficient** | 0.134 | -0.024 | 0.112 | -0.017 |
| **SD** | 0.035 | 0.064 | 0.040 | 0.004 |

Each location was assigned the *HydroBasins* sub-basin it is located in at Pfafstetter level 7 by the minimum Euclidian distance from the location to the polygon outline (< 0 if the location is inside the specific polygon). Point data located outside of any sub-basin was excluded from further analysis, as these tend to be located in estuaries or even coastal regions, where marine influence becomes significant. The mean of annual averages of all locations within the same subbasin was calculated (mostly 2 locations per sub-basin).
The average lithological composition for each sub-basin and respective upstream values were calculated using the *SAGA* toolbox (Conrad et al., 2015) in the open-access geographic information system *QGIS* (QGIS Development Team, 2021). For this purpose, the Global Lithological Map (*GLiM)* was converted to WGS 84, vectorized and polygons were dissolved by lithological class. The absolute and relative lithological composition of each sub-basin was calculated using the area occupied by each mono-lithological unit within that sub-basin. Upstream values were then calculated for each sub-basin from all basins indicated to flow into the specific basin by following the NEXT_DOWN identifier in the nested HydroBasins catchment outlines for a maximum of 420 basins back, exceeding the longest flow path at Pfaffstetter level 7. To combine this lithological information into a single variable, we assigned each lithological and unconsolidated sediment unit a source carbonate content in accordance to the descriptions provided in the respective reference (Börker et al., 2018; Hartmann and Moosdorf, 2012). Carbonate sediments were assumed to purely consist of carbonate, mixed sediments were assumed a 50:50 mixture of siliciclastic and carbonate sediments and one third of all evaporites was assumed to be carbonate. The carbonate content of metamorphic rocks can range from 0 to 100 and its average is unknown on a global scale. We therefore assume the composition of metamorphic rocks to be equal to the average composition of the rest of Earth’s rock surface, yielding a carbonate content of 27 % (Hartmann and Moosdorf, 2012). Procedures were followed similarly for the map of unconsolidated sediments (*GUM*, Börker et al., 2018), to assign globally representative carbonate concentrations to each sediment class using published global estimates (Journet et al., 2014; Müller et al., 2021b) and the *GeoReM* geomaterial reference database (Jochum et al., 2005). The unconsolidated sediment unit (‘su’) in *GLiM* was assigned no carbonate content to avoid overlap with the *GUM*. Details of the assignment can be inspected in the according scripts (supplementary MATLAB file ‘PredictorSets.m’ and ‘MapUnit_CarbonateContent’.xlsx’). For soils, the carbonate content was given directly as a polygon property within the database (WISE, Batjes, 2012). All three carbonate sources were summed, normalized to 100% and used as an indication of the potential source carbonate (SC), i.e., the carbonate available to be transported as PIC by the respective river.
Notably, matrix carbonate can be present in nominally carbonate-free siliciclastic sediments and accounted for by assigning 10 % carbonate to this unit (Figure S 3). This assignment neglects the chemical and mineralogical heterogeneity of the individual rock units. For instance, alluvial sediment may be extremely diverse and not well-represented by a global average. This becomes visible in high PIC values (several wt%) that were assigned a low SC (< 10 %), which is implausible because of the dissolution-dominated weathering behavior of carbonates. While we notice that this issue might be the major source of inaccuracy of our results, these values (where River PIC > 0.8*SC-related PIC) were excluded from our analysis as they rather reflect a misfit of the maps used, our carbonate assignment and the PIC concentrations, then the true relative variance of the variables.
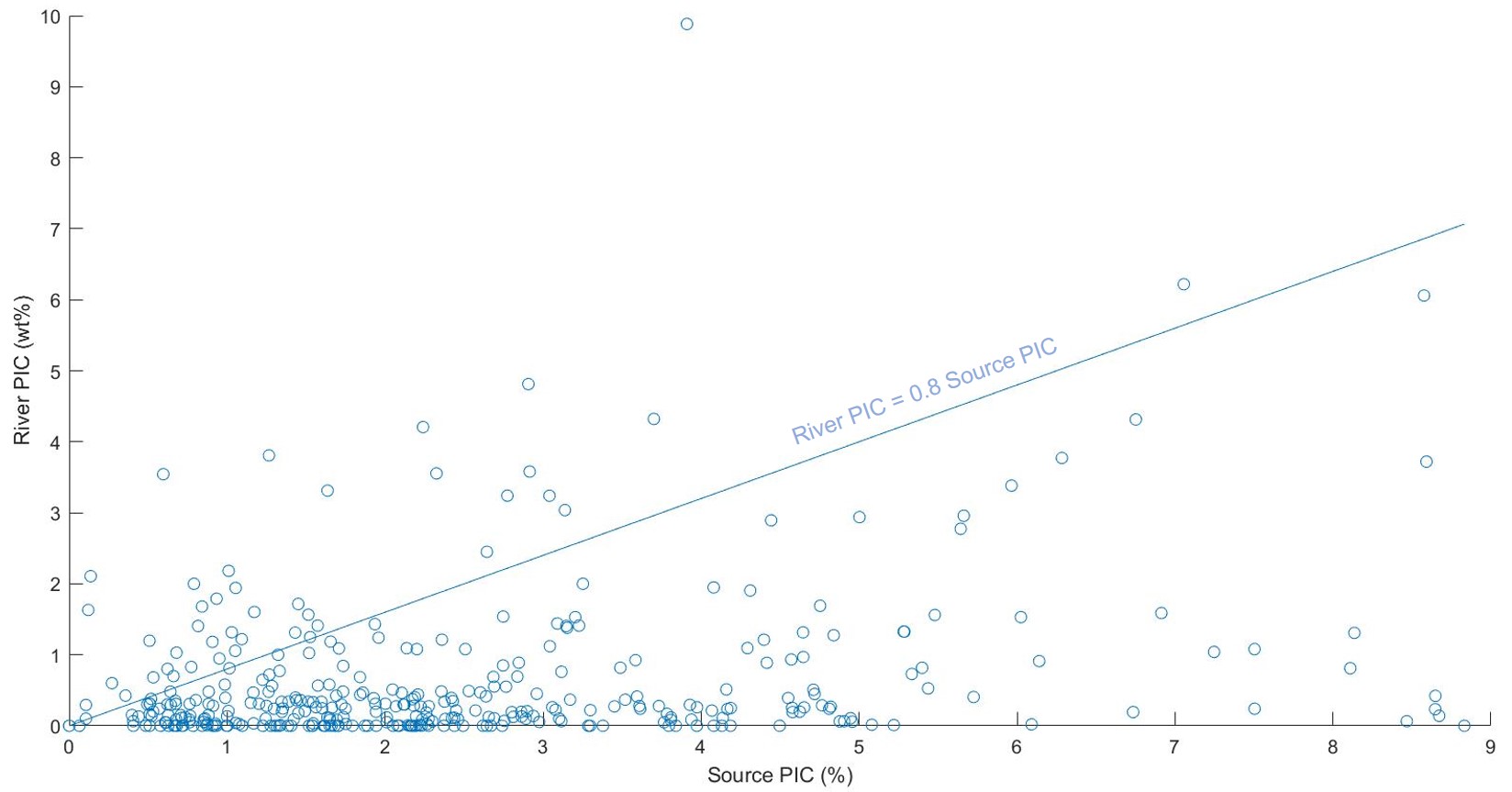
 **Figure S 3** PIC vs source carbonate content from rocks, sediments and soils within the respective catchment. The blue line represents our logical constraint that river PIC < 0.8 times the source carbonate related PIC.

The value of 0.8 is somewhat arbitrarily chosen, but considering 0.9 or 0.7 makes negligible differences and the main point is to remove unreasonably high PIC values (compared to the maps used). The 297 variables provided by *HydroBasins* were reduced to 99 variables by selecting only upstream The main point is to remove unreasonably high PIC values (in respect to the maps used).and annual averages. From these 99 variables, potentially important predictors were identified and grouped using the correlation matrix and established causal relationships to PIC and carbonate weathering. For highly intercorrelated variables, e.g. precipitation and runoff, only the one was selected that better correlated to PIC and cannot be represented by any other variable or variable combination (e.g., runoff by precipitation and upstream area). The 9 selected variables are presented in the main text

## S2 Regression and Upscaling

A justification of the chosen approach to regression and general information are given in the main text, while in the following the exact setting and procedures of the regression are described in detail.

**S2.1 Qualitative indication of PIC presence**

For the qualitative prediction of PIC presence (yes/no), we assigned each location in our data compilation a class (0 = no, 1 = yes) according to a critical threshold concentration of 0.1 wt% PIC, that can effectively not be distinguished from zero within uncertainty. 75 % of the dataset were used to train a set of different models derived from employing different methods, such as linear and logistic regression, tree- and ensemble techniques, discriminant analysis, nearest neighbor, naïve Bayes classifiers and Support Vector Machines (SVM), all of which are available in the MATLAB 2019b Machine Learning toolbox. Of those, Ensemble techniques, naïve Bayes classifiers and SVMs performed similarly well with respect to the accuracy of positive indications, while only SVMs exhibit the same quality for negative indications. We therefore chose to (automatically) optimize the hyperparameters of an SVM algorithm with respect to our dataset, yielding a final accuracy of 83.6 % for positive classifications and of 77 % for negative classification. For the negative indications, we inserted LHS-distributed numbers within the range of 0 to 0.1 wt% in the Monte Carlo simulation. In the end, the uncertainty on our global estimate that is induced by SVM accuracy is potentially very small, because even false positive indications may result in PIC values close to the truth. The reason is that the quantitative model would tend to still predict low values in these cases. False negative indications, in contrast, may actually scratch the lower limit of what is reliably predictable, but are likely close to a value within a range of 0 to 0.2 wt%, as observed in individual test plots of the quantitative prediction.

**S2.2 Quantitative symbolic regression by Multi-Gene Genetic Programming**

The open-source toolbox GPTIPS 2.0 (Searson et al., 2010) was implemented for Symbolic Regression based on Multi-Gene Genetic Programming. Due to changes in the MATLAB symbolic math toolbox, GPTIPS can currently only be used in MATLAB versions older than v2018a. Symbolic Regression by GPTIPS was used to derive the form and the parameters of a quantitative model to predict PIC concentrations from catchment properties. Single terms (‘Genes’) are (pseudo-)randomly created along tree-like structures and linearly combined to full models by weighting coefficients estimated using classic least-squares regression. These models are applied to the training subset and results are compared to observed PIC concentrations in individual ‘tournaments’ of pre-defined size. The best performing models in these tournaments are evolved through genetic operations, such as numerical mutation, copy or exchange (‘cross-over’).
Apart from the chosen predictor variables and constants between -10 and 10, individual genes can contain the following operators/functions: times, plus, minus, power, exp(), log(), square root(), exp(*-1), *-1, tanh() and cosh(). Each run starts with a? population of? 1000 models consisting of up to 5 genes with a maximum tree-depth of 3. In a single run, equations were evolved over 500 generations with a 20 % probability of mutation by a Gaussian function with a standard deviation of 0.2, 78 % probability of cross-over (of which 30 % are high-level cross-overs) and 2 % of direct copy. Tournament size was set to 2, comparing a maximum of models to each other. To reduce complexity of the resulting equations, a pareto-fraction of 10 % was introduced, that is 10 % of the tournament decisions include a penalty for expressional complexity (= sum of number of nodes in all sub-trees describing a model). For the rest, our fitness function was defined as the root mean squared error (RMSE):

$Fitness= RMSE=\sqrt{\frac{\sum_{i}^{N} {(\mathrm{Obs}_{i}-\mathrm{Pred}_{i})}^{2}}{N}}$ (S2)

Where *N* is the number of samples, *i* is an integer between 0 and *N*, *Obs* is the observed value and *Pred* the prediction. To avoid overfitting, each model was tested on a 25 % subset, that was not considered during training. The model that performed best compared to this test set was chosen as the output.

**S 2.3 Calculation of the global riverine PIC flux and Monte Carlo Simulations**

The result of the data compilation and regression efforts is a gapless composite set of observed, modelled and zero-constrained PIC concentrations for each *HydroBasins* (sub-)basin at Pfafstetter level 7. For the basins directly draining into the ocean, PIC concentrations were multiplied by their respective sediment discharge to yield PIC fluxes, which were then summed up to yield the global riverine PIC flux. To test for the effect of anthropogenic reduction of the global sediment discharge (Cohen et al., 2014), we repeated this procedure using disturbed sediment discharge values provided by *GlobalDelta* (Caldwell et al., 2019; Nienhuis et al., 2020).
The exact number resulting from this procedure would change, if (i) different subsets would have been selected for the individual runs, (ii) different samples would have been available (uncertainty of annual averages and representativity of the input data), (iii) another sediment flux would have been implemented (uncertainty of the sediment discharge model). The influence of the accuracy of the input maps is not considered in the error analysis and discussed below. To account for the effect such variations exert on our estimation of global PIC flux (i-iii), we re-estimated the PIC flux as the mean of 830 Monte Carlo simulations (MC), while the associated uncertainty is represented by the standard deviation of the individual results (Koehler et al., 2009). The number of simulations was determined by monitoring the change in mean values and standard deviations (Koehler et al., 2009), which appear to stabilize within a few 100 repetitions. The 830 simulation used were selected from a total of 2,000 simulations based on the frequency of outliers in respect to the 10 % and 90 % percentile of all 2,000 results for the same basin. The 830 simulations selected had < 0.3 % outliers in PIC fluxes.

In each MC simulation, observed PIC concentrations were perturbed by a pseudo-random factor between -0.3 and 0.3, which corresponds to the mean relative uncertainty of the annual averages. Evenly distributed perturbation factors were achieved using LHS. This implies that our uncertainty estimate is an upper limit, because even distributions facilitate more variability than Gaussian distribution through higher frequencies of marginal values. Subsequently, the full regressive classification and symbolic regression procedure was repeated for each of these perturbed sets and predictions were made for the missing basins with a positive indication of PIC presence. Basins with SC < 10 % were manually set to 0 for all runs (see 2 Methods, Main Text). Finally, the individual simulations were combined with a set of sediment discharge perturbed by an LHS-based factor between – 0.5 and 0.5. This range is oriented on the performance of the *WBMSed* 2.0 model at a number of well-distributed test locations (Cohen et al., 2014) and on the range of published estimates of the global sediment discharge of ~ 12 – 20 Gt/y (e.g., Beusen et al., 2005; Milliman and Farnsworth, 2011; Syvitski and Kettner, 2011). For each individual basin, outliers between the accepted simulations and unreasonable high values (> 12 wt% PIC corresponds to > 100 wt% carbonate) were removed before further statistical treatment.

This extensive uncertainty analysis accounts for all variations on our results, except for the accuracy of the maps our model is based on. This is particularly true for the derivation of SC, which strongly depends on the assumptions made during carbonate assignment to the individual units, especially those of unconsolidated sediment. The accuracy of the lithological map (*GLiM*) was estimated to ~ 60 %, considering nominally different, but similar units as a fit (Hartmann and Moosdorf, 2012). This demonstrates the general issue, that modelling of Earth-system processes can only be as good as we observe and record Earths’ properties. Variables related to human influence may also be subjected to different sorts of bias, while records of temperature, precipitation, soil carbonate, the extent of water bodies and the rough indications of vegetation type seem more reliable, as these are more easily measured or documented. However, there may also be a degree of uncertainty emerging from the extrapolation and harmonization procedures used to generate the available gridded and gapless maps that we used as input variables (Linke et al., 2019).

Additionally, the importance of individual variables for the prediction of PIC concentrations was evaluated by the coefficients of correlation and determination between each variable and the modelled Monte Carlo-mean PIC concentrations (see main text). This method reduces biases due to multi-collinearity and non-linearity and is commonly applied to the evaluation of canonical correlations analyses (Kuylen and Verhallen, 1981).

## S3 First-order estimates

Literature data for the average river PIC concentration (relative to dry suspended sediment mass) globally range from 1 wt% (Meybeck, 1982) to 4 wt% (Savenko, 2007), compared to 0.4 wt% for the continental USA (Canfield, 1997). Despite denoted as inorganic carbon, we assume the second value (Savenko, 2007) to represent the carbonate concentration rather than the carbon concentration. The conversion to mass % of carbon by the multiplication with 0.2 (20 % carbon in CO_3_) yields 0.8 wt%, which is in the range of the other estimates. Considering a global riverine sediment flux of 19.1 Pg/y (Beusen et al., 2005; Cohen et al., 2014; Milliman and Farnsworth, 2011) with 21 % being related to (nominally) carbonate-free basaltic lithologies (Milliman and Syvitski, 1992), the global PIC flux might be 63 to 150 Mt C/y ( = 5 to 12.5 Tmol C/y). This is slighlty lower than the value of Gattuso et al. (1998) (14 Tmol C/y), but consistent with the range reported by Middelburg et al. (2020) (7.4-14.2 Tmol C/y).

From the difference between the global mean major element composition of riverine suspended particles with and without decarbonation during sample preparation (Bayon et al., 2015; Viers et al., 2009), we estimate a global average PIC concentration of 0.6 wt% based on a concentration of carbonate-related CaO of 2.9 wt% and a C/Ca ratio of 0.21 in calcite. This results in a PIC flux of 92 Mt C/y or 12.7 Tmol C/y, consistent with the previously estimated range. These first-order estimates support our hypothesis, that riverine PIC fluxes may indeed be a significant, but largely overlooked flux in the Earth system. Notably, these concentrations are not flux-weighted and based on rather small and incomprehensible datasets. As tropical rivers dominate the global sediment flux to the ocean by far (Milliman and Farnsworth, 2011) and exhibit the highest rates of chemical weathering (Hartmann et al., 2014) and carbonate dissolution (Romero-Mujalli et al., 2019), the true magnitude of the global PIC flux and its behavior in response to changing sound conditions remain very uncertain.

Greenland and Antarctica were not included in the model estimates, as they are not part of the sediment discharge model (Cohen et al., 2014), and are hardly mapped. We therefore took published estimates of sediment discharge by meltwater and ice-rafted debris from literature (Overeem et al., 2017; Raiswell et al., 2008; Wadham et al., 2013). We further assume that the PIC concentration of sediment discharged from Greenland and Antarctica is equal to the average global concentration, which is in line with the few available measurements from Greenland available in *GloRiSe* v1.1 (Müller et al., 2021b). The average PIC concentration of atmospheric dust was taken from a model-estimate of the global average dust composition (Journet et al., 2014) and complemented by a widely accepted estimate for atmospheric dust deposition in the ocean (Jickells et al., 2005). Numbers and calculus can be found in the supplementary files (‘CryosphereAtmosphere.xslx’).

## Data and script availability

The all data and scripts used for this study, along with a detailed manual and the supplementary information, can be accessed via: <https://doi.org/10.5281/zenodo.6125880> (DOI: 10.5281/zenodo.6125880).

## References

Batjes, N. H. (2012). *ISRIC-WISE derived soil properties on a 5 by 5 arc-minutes global grid (ver. 1.2)*. ISRIC - World Soil Information.

Bayon, G., Toucanne, S., Skonieczny, C., André, L., Bermell, S., Cheron, S., et al. (2015). *Rare earth elements and neodymium isotopes in world river sediments revisited*. *Geochimica et Cosmochimica Acta* (Vol. 170). https://doi.org/10.1016/j.gca.2015.08.001

Beusen, A. H. W., Dekkers, A. L. M., Bouwman, A. F., Ludwig, W., & Harrison, J. (2005). Estimation of global river transport of sediments and associated particulate C, N, and P. *Global Biogeochemical Cycles*, *19*(4). https://doi.org/10.1029/2005GB002453

Börker, J., Hartmann, J., Amann, T., & Romero-Mujalli, G. (2018). Terrestrial sediments of the earth: Development of a global unconsolidated sediments map database (gum). *Geochemistry, Geophysics, Geosystems*, *19*(4), 997–1024. https://doi.org/10.1002/2017GC007273

Caldwell, R. L., Edmonds, D. A., Baumgardner, S., Paola, C., Roy, S., & Nienhuis, J. H. (2019). A global delta dataset and the environmental variables that predict delta formation on marine coastlines. *Earth Surface Dynamics*, *7*(3), 773–787. https://doi.org/10.5194/esurf-7-773-2019

Canfield, D. E. (1997). The geochemistry of river particulates from the continental USA: Major elements. *Geochimica et Cosmochimica Acta*, *61*(16), 3349–3365. https://doi.org/10.1016/S0016-7037(97)00172-5

Cohen, S., Kettner, A. J., & Syvitski, J. P. M. (2014). Global suspended sediment and water discharge dynamics between 1960 and 2010: Continental trends and intra-basin sensitivity. *Global and Planetary Change*, *115*, 44–58. https://doi.org/10.1016/j.gloplacha.2014.01.011

Conrad, O., Bechtel, B., Bock, M., Dietrich, H., Fischer, E., Gerlitz, L., et al. (2015). System for Automated Geoscientific Analyses (SAGA) v. 2.1.4. *Geoscientific Model Development*, *8*(7), 1991–2007. https://doi.org/10.5194/gmd-8-1991-2015

Gattuso, J. P., Frankignoulle, M., & Wollast, R. (1998). Carbon and Carbonate Metabolism in Coastal Aquatic Ecosystems Author ( s ): J . -P . Gattuso , M . Frankignoulle and R . Wollast Source : Annual Review of Ecology and Systematics , Vol . 29 ( 1998 ), pp . 405-434 Published by : Annual Reviews Stable URL : *Annual Reviews in Ecology and Systematics*, *29*(1998), 405–434.

Hartmann, J., & Moosdorf, N. (2012). The new global lithological map database GLiM: A representation of rock properties at the Earth surface. *Geochemistry, Geophysics, Geosystems*, *13*(12), 1–37. https://doi.org/10.1029/2012GC004370

Hartmann, J., Moosdorf, N., Lauerwald, R., Hinderer, M., & West, A. J. (2014). Global chemical weathering and associated p-release - the role of lithology, temperature and soil properties. *Chemical Geology*, *363*, 145–163. https://doi.org/10.1016/j.chemgeo.2013.10.025

Jickells, T. D., An, Z. S., Andersen, K. K., Baker, A. R., Bergametti, C., Brooks, N., et al. (2005). Global iron connections between desert dust, ocean biogeochemistry, and climate. *Science*, *308*(5718), 67–71. https://doi.org/10.1126/science.1105959

Jochum, K. P., Nohl, U., Herwig, K., Lammel, E., Stoll, B., & Hofmann, A. W. (2005). GeoReM: A new geochemical database for reference materials and isotopic standards. *Geostandards and Geoanalytical Research*, *29*(3), 333–338. https://doi.org/10.1111/j.1751-908x.2005.tb00904.x

Journet, E., Balkanski, Y., & Harrison, S. P. (2014). A new data set of soil mineralogy for dust-cycle modeling. *Atmospheric Chemistry and Physics*, *14*(8), 3801–3816. https://doi.org/10.5194/acp-14-3801-2014

Koehler, E., Brown, E., & Haneuse, S. J.-P. A. (2009). On the Assessment of Monte Carlo Error in Simulation-Based Statistical Analyses. *Am. Stat.*, *63*(2), 1-155–162. https://doi.org/10.1198/tast.2009.0030.On

Kuylen, A. A. A., & Verhallen, T. M. M. (1981). The use of canonical analysis. *Journal of Economic Psychology*, *1*(3), 217–237. https://doi.org/10.1016/0167-4870(81)90039-8

Linke, S., Lehner, B., Ouellet Dallaire, C., Ariwi, J., Grill, G., Anand, M., et al. (2019). Global hydro-environmental sub-basin and river reach characteristics at high spatial resolution. *Scientific Data*, *6*(1), 283. https://doi.org/10.1038/s41597-019-0300-6

Meybeck, M. (1982). Carbon, nitrogen, and phosphorus transport by world rivers. *American Journal of Science*. https://doi.org/10.2475/ajs.282.4.401

Milliman, J. D., & Syvitski, J. P. M. (1992). Geomorphic/tectonic control of sediment discharge to the ocean: the importance of small mountainous rivers. *Journal of Geology*, *100*(5), 525–544. https://doi.org/10.1086/629606

Milliman, John D., & Farnsworth, K. L. (2011). *River discharge to the coastal ocean: A global synthesis*. *River Discharge to the Coastal Ocean: A Global Synthesis*. https://doi.org/10.1017/CBO9780511781247

Müller, G., Middelburg, J. J., & Sluijs, A. (2021a). Global River Sediments (GloRiSe). Zenodo. https://doi.org/10.5281/zenodo.4447435

Müller, G., Middelburg, J. J., & Sluijs, A. (2021b). Introducing GloRiSe - A global database on river sediment composition. *Earth System Science Data*, *13*, 3565–3575. https://doi.org/https://doi.org/10.5194/essd-13-3565-202

Nienhuis, J. H., Ashton, A. D., Edmonds, D. A., Hoitink, A. J. F., Kettner, A. J., Rowland, J. C., & Törnqvist, T. E. (2020). Global-scale human impact on delta morphology has led to net land area gain. *Nature*, *577*(7791), 514–518. https://doi.org/10.1038/s41586-019-1905-9

Overeem, I., Hudson, B. D., Syvitski, J. P. M., Mikkelsen, A. B., Hasholt, B., Van Den Broeke, M. R., et al. (2017). Substantial export of suspended sediment to the global oceans from glacial erosion in Greenland. *Nature Geoscience*, *10*(11), 859–863. https://doi.org/10.1038/NGEO3046

QGIS Development Team. (2021). QGIS Geographic Information System. Retrieved from http://qgis.org

Raiswell, R., Benning, L. G., Tranter, M., & Tulaczyk, S. (2008). Bioavailable iron in the Southern Ocean: The significance of the iceberg conveyor belt. *Geochemical Transactions*, *9*, 1–9. https://doi.org/10.1186/1467-4866-9-7

Romero-Mujalli, G., Hartmann, J., & Börker, J. (2019). Temperature and CO2 dependency of global carbonate weathering fluxes – Implications for future carbonate weathering research. *Chemical Geology*, *527*(July), 118874. https://doi.org/10.1016/j.chemgeo.2018.08.010

Savenko, V. S. (2007). Chemical composition of sediment load carried by rivers. *Geochemistry International*, *45*(8), 816–824. https://doi.org/10.1134/S0016702907080071

Searson, D. P., Leahy, D. E., & Willis, M. J. (2010). GPTIPS:An open source genetic programming toolbox for multigene symbolic regression. *Proceedings of the International MultiConference of Engineers and Computer Scientists 2010, IMECS 2010*, *I*, 77–80.

Syvitski, J. P. M., & Kettner, A. (2011). Sediment flux and the anthropocene. *Philosophical Transactions of the Royal Society A: Mathematical, Physical and Engineering Sciences*, *369*(1938), 957–975. https://doi.org/10.1098/rsta.2010.0329

Viers, J., Dupré, B., & Gaillardet, J. (2009). Chemical composition of suspended sediments in World Rivers: New insights from a new database. *Science of the Total Environment*, *407*(2), 853–868. https://doi.org/10.1016/j.scitotenv.2008.09.053

Wadham, J. L., De’Ath, R., Monteiro, F. M., Tranter, M., Ridgwell, A., Raiswell, R., & Tulaczyk, S. (2013). The potential role of the Antarctic Ice Sheet in global biogeochemical cycles. *Earth and Environmental Science Transactions of the Royal Society of Edinburgh*, *104*(1), 55–67. https://doi.org/10.1017/S1755691013000108
